# How to Build a Microplastics‐Free Environment: Strategies for Microplastics Degradation and Plastics Recycling

**DOI:** 10.1002/advs.202103764

**Published:** 2022-01-06

**Authors:** Junliang Chen, Jing Wu, Peter C. Sherrell, Jun Chen, Huaping Wang, Wei‐xian Zhang, Jianping Yang

**Affiliations:** ^1^ State Key Laboratory for Modification of Chemical Fibers and Polymer Materials College of Materials Science and Engineering Donghua University Shanghai 201620 China; ^2^ Co‐Innovation Center for Textile Industry Innovation Center for Textile Science and Technology Donghua University Shanghai 201620 China; ^3^ Department of Chemical Engineering The University of Melbourne Parkville Victoria 3010 Australia; ^4^ ARC Centre of Excellence for Electromaterials Science Intelligent Polymer Research Institute (IPRI) Australian Institute of Innovative Materials (AIIM) University of Wollongong Wollongong New South Wales 2522 Australia; ^5^ College of Environmental Science and Engineering State Key Laboratory of Pollution Control and Resources Reuse Tongji University Shanghai 200092 P. R. China

**Keywords:** catalytic conversion, degradation, microplastics, plastic waste, recycle

## Abstract

Microplastics are an emergent yet critical issue for the environment because of high degradation resistance and bioaccumulation. Unfortunately, the current technologies to remove, recycle, or degrade microplastics are insufficient for complete elimination. In addition, the fragmentation and degradation of mismanaged plastic wastes in environment have recently been identified as a significant source of microplastics. Thus, the developments of effective microplastics removal methods, as well as, plastics recycling strategies are crucial to build a microplastics‐free environment. Herein, this review comprehensively summarizes the current technologies for eliminating microplastics from the environment and highlights two key aspects to achieve this goal: 1) Catalytic degradation of microplastics into environmentally friendly organics (carbon dioxide and water); 2) catalytic recycling and upcycling plastic wastes into monomers, fuels, and valorized chemicals. The mechanisms, catalysts, feasibility, and challenges of these methods are also discussed. Novel catalytic methods such as, photocatalysis, advanced oxidation process, and biotechnology are promising and eco‐friendly candidates to transform microplastics and plastic wastes into environmentally benign and valuable products. In the future, more effort is encouraged to develop eco‐friendly methods for the catalytic conversion of plastics into valuable products with high efficiency, high product selectivity, and low cost under mild conditions.

## Graphical abstract

The emerging microplastics issue has received enormous amount of attentions recently as the adverse impacts on living things. The developments of catalytic conversion of microplastics and plastic wastes into harmless matters and valuable chemicals give the promising route for not only building a microplastics‐free environment but also constructing the circular economy mode.

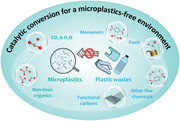

## 1 Introduction

**Figure 1:**
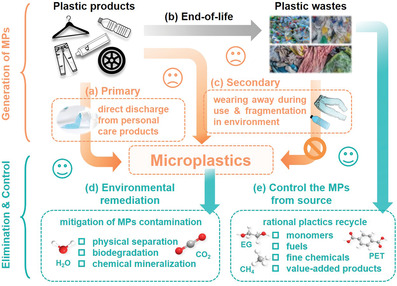
Pathways of the generation of MPs and concept to build a MPs free environment. a) Generation of primary MPs. b) The end‐of‐life of plastic products. c) Generation of secondary MPs plastic wastes. d) Chemical degradation of the existing environmental MPs. e) Rational plastic wastes managements to alleviate the generation MPs from the key source.

Microplastics (MPs), defined as plastic debris with diameters smaller than 5 mm,^[^ ^1^, ^2^ ^]^ are regarded as an emerging environmental contaminant and have received enormous amounts of attention due to their potential adverse impacts on living things. Depending on their sources, MPs are classified as primary and secondary MPs. As shown in **Figure** **1**, primary MPs are plastic particles designed and produced intentionally for a given application, such as, micro‐sized plastic microspheres, fragments, and microfibers pervasively applied in personal care products or synthetic textiles. In contrast, secondary MPs are unintentionally formed from the gradual fragmentation of mismanaged plastic wastes by photolysis, abrasion, and/or microbial decomposition. Whether intentionally or unintentionally produced, MPs with varied shapes, including beads, foams, fibers, and films,^[^ ^3^, ^4^, ^5^ ^]^ have been detected in air,^[^ ^6^ ^]^ aquatic systems,^[^ ^7^, ^8^, ^9^, ^10^ ^]^ river and ocean sediments,^[^ ^11^ ^]^ and soil.^[^ ^3^, ^12^ ^]^ The fast release of MPs with their combined high resistance against degradation results in a rapid accumulation of these particles in the natural environment.^[^ ^13^, ^14^ ^]^ It is predicted that the total mass of plastic debris cumulated in ocean could increase to ≈250 million metric tons (Mt) by 2025, which is an order of magnitude higher than in 2010.^[^ ^15^ ^]^ The light and small nature of MPs lead to their easy transportation with wind and water flow in environment. As a consequence, MPs have been found worldwide both close to human habitation and in remote areas far away from human activities. After long‐term exposure to MPs, the chronic toxicity including impaired reproduction and malnutrition can be caused, posing a threat to biota and humans. Further, due to the relatively large specific surface area of MPs, heavy metals,^[^ ^16^ ^]^ and persistent organic pollutants (POPs)^[^ ^17^, ^18^ ^]^ are prone to adhere and accumulate on the surface of MPs and then migrate in environment. It has been reported that the concentrations of POPs adsorbed onto the MPs can be 10^[^ ^6^ ^]^ higher than that in the ambient environment.^[^ ^19^ ^]^ Thus, further transfer and accumulation of these hazardous POPs carried MPs in the food chain lead to serious potential threats to human health. The level of concern around MPs is so great, governments globally are legislating against the production of primary MPs.^[^ ^20^ ^]^

**Figure 2:**
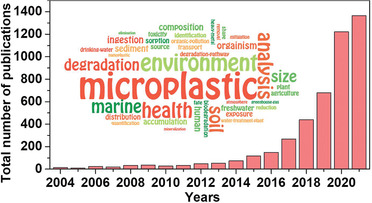
The number of annual publications and keywords (insert) on MPs research in Web of Science Core Collection (WoSCC) database from 2004 to November of 2021. (Insert created using wordle.net.)

To understand the development trends and subject areas of research related to MPs, we analyzed the publications published from 2004, when the concept of microplastics was first proposed by Thompson et al.^[^ ^21^ ^]^ to November of 2021 by using “microplastic” or “micro‐plastic” or “micro‐sized plastic” as keywords. We obtained 4692 records from Web of Science Core Collection (WoSCC) database. As shown in **Figure** **2**, the number of the published papers on MPs research have increased exponentially in the last 10 years, and about 65.72% (3261 items) of the total publications were published in the most recent 3 years, reflecting that this topic is of critical importance.

The articles (83.65%) and reviews (10.17%) account for the majority of the papers (a sum of 4402 papers). We summarized the most prevalent keywords from these papers and the insert of Figure 2 illustrates the frequency visualization of keywords among these identified papers. The larger size of these keywords indicates that the more papers have been published concerning such topics. The most frequently used keywords are microplastics, health, environment, marine, analysis, degradation, and soil. There are many high‐quality reviews focusing on the hot spots mentioned above. Ganesh et al. reported an overview of degradability of typical synthetic plastics in the marine environment and highlighting the degradation resistance of MPs and their long‐term toxic effects on marine organisms.^[^ ^22^ ^]^ Ivleva et al. reviewed the methods of analysis for aquatic MPs, uptake of MPs in aquatic organisms, and toxicity effects on these aquatic organisms.^[^ ^1^ ^]^ Elkhatib et al. compared the sampling, processing, identification and quantification technologies of MPs in several studies.^[^ ^23^ ^]^ Chamas et al. discussed the environmental effects on degradation rates of MPs with respect to their shape, size, and chemical composition.^[^ ^24^ ^]^ Guo et al. gave an overview on the MPs’ sorption behaviors on heavy metals and POPs in marine environment.^[^ ^25^ ^]^ In previous studies, researchers focused on the understanding of the characterization of MPs in natural environments especially in marine and soil, as well as, their impacts to health of living things. Taking into account the rapid accumulation of MPs and severe contamination of these pollutants in the environment, pioneering researchers began to emphasize the importance of developing efficient strategies to mitigate MPs contamination. Even though researchers have discovered the insufficient nature of traditional technologies in practical application for MPs removal, reviews mostly focused on summarizing traditional physical methods rather than discussing the potential alternatives and their challenges. Meanwhile, the mismanaged plastic wastes in environment are also worth considering, since they can degrade and gradually fragmented into MPs. Physical separation via waste water treatment plants (WWTPs) remains the primary mechanism of removing MPs. Unfortunately, the sludge from physical separation is often recycled for landfill or agricultural applications, leading to MPs re‐entering the water system. Taking the sludge used for agriculture in North America as an example, there are 4.4 × 10^4^ to 3.0 × 10^5^ tons of MPs that return to environment per year.^[^ ^26^ ^]^ Subsequently, MPs can return to water systems again through soil erosion or surface runoff. Thus, emerging methods that permanently degrade and remove MPs are critical to remediating the environment. Hence, it is an urgent need to review the recent advanced technologies for transformation of MPs into non‐toxic matter or recycling plastic wastes into valuable products. On the other hand, researchers proved the relationship between MPs and plastic wastes that plastic wastes in landfills and environment are potential sources for MPs releasing under weathering, light radiation, and microorganisms.^[^ ^27^, ^28^, ^29^ ^]^ Unfortunately, less than 9% of the total plastic wastes were recycled due to the high cost, low productivity of current recycle approaches. Thus, the development of efficient and green methods to catalytic convert plastic wastes into valorized chemicals is significant, since it can not only prevent the release of MPs but also reclaim raw materials for plastics industry. In summary, the two strategies jointly contribute to create a green and sustainable MPs‐free environment by eliminating MPs existing in environment and preventing the generation of MPs from the significant potential source.

In this critical review, we take a holistic approach to managing, mitigating, and remediating MPs from the environment. First, we highlight the recent innovations on transforming MPs into environmental‐friendly products and reveal their underlying mechanisms and challenges to directly alleviate the potential health risk for living organisms. Second, we focus on the recycling strategies of plastic wastes, which is highly important to suppress the release of secondary MPs by potentially yielding new fine chemicals or fuels. Novel catalytic methods such as, photocatalysis, catalytic advanced oxidation process (AOP), and biodegradation (enzymatic catalysis) open the door to convert MPs and plastic wastes into non‐toxic products or valuable products (monomers, fuels, or other value‐added fine chemicals). Advanced catalyst design is significant to promote the development of these methods, where the product selectivity, conversion efficiency, reaction rate can be further optimized. The overall aim of this review is to inspire innovation for green and efficient catalytic strategies targeting on MPs contamination elimination and plastic wastes recycling to comprehensively build a MPs‐free environment.

## 2 Sources, Fate, and Impacts of Microplastics

Plastics are high molecular weight synthetic polymers produced by polymerization of various types of monomers derived mainly from fossil feedstocks. Nowadays, plastics are an essential type of material in our society due to their light‐weight, relatively low price, excellent moldability, and durability. The global production of plastics continuously increases during the last decades and this figure increased to 367 Mt in 2020 from 2 Mt in 1950.^[^ ^30^ ^]^ However, the overuse of plastics has caused serious MPs contamination.

Primary MPs are continuously released into nature through the use of micro‐sized plastics‐contained products in our daily lives. For instance, polyethylene (PE) particles with size between 70 and 400 µm have been frequently used as exfoliators in facial cleaners and toothpaste.^[^ ^31^ ^]^ About 0.74 to 4.8 g of PE microbeads (diameters smaller than 1000 µm) were added to 100 mL of body and facial scrub, which result in 15.2 mg MPs release into the sewage system per person per day.^[^ ^32^ ^]^ Laundry of textiles based on polyester (PET) polyamide (PA) and other kinds of chemical microfibers has also been known as an important source of primary MPs as well. It was reported that >1900 fiber‐based MPs were produced by washing of a single garment.^[^ ^2^ ^]^

The release of secondary MPs is mostly derived from the littered plastic wastes. Based on the lifetime statistics, the majority of plastic products are used for short‐term applications. About 40% of plastic products have a lifetime shorter than one month or for disposable uses. To date, the cumulative mass of virgin plastics on the earth has reached to 8300 Mt, with 6300 Mt ending up as waste.^[^ ^33^ ^]^ Unfortunately, only 9% and 12% of the total plastic wastes end up in recycle and incineration, while 79% of plastic wastes have been landfilled or directly enter in nature environment.^[^ ^34^ ^]^ Landfilling with characteristic of facility was considered as major option for plastic wastes managements in the past years. Geyer et al. estimated that the sum of plastic wastes accumulated in landfills or natural environment globally were around 4900 Mt (60% of all plastics ever produced) and this number will reach to roughly 12 000 Mt by 2050.^[^ ^33^ ^]^ Although it seems that the landfill treatment is the easiest and most economical method, the buried plastic waste can lead to greater impacts by constantly releasing secondary MPs due to several physical or chemical degradation effects. The chemical composition of secondary MPs is more various than primary MPs, since many categories of plastics are used in our lives. Secondary MPs can be released by wearing of tires (synthetic rubber), which can be carried by stormwater runoff and enter water systems.^[^ ^35^ ^]^ The use and disposal of PE, PA, and polypropylene (PP) fibers during fishing and other water activities also release a large amount of secondary MPs.^[^ ^36^ ^]^ Meanwhile, the chemical composition of secondary MPs will change during weathering, since the generation of oxygen‐containing groups and unsaturation bonds on the polymer chain.^[^ ^24^ ^]^ As the result, the slightly changed density and hydrophilicity of secondary MPs can influence the MPs removal efficiency in WWTPs, which is discussed in later sections. Due to the breakdown of plastics triggered by physical wear and chemical degradation, secondary MPs possess wider size distribution with the range of millimeters to nanometers. It has been reported that numerous smaller‐sized MPs (<500 µm) and even nanoplastics (NPs, <1 µm) could be released during the secondary degradation and fragmentation of primary and secondary MPs.^[^ ^13^, ^37^, ^38^ ^]^ Even worse, compared with bulk plastics, smaller‐sized MPs are more difficult to clean and more hazardous.^[^ ^39^, ^40^, ^41^, ^42^ ^]^

Water systems are the mostly heavily polluted area by MPs.^[^ ^15^ ^]^ Su et al. reported that the abundance of MPs in the surface water samples of Taihu Lake is about 6.80 × 10^6^ items km^−2^, which is the highest among all the known data about freshwater lakes worldwide.^[^ ^43^ ^]^ Fibrous microplastics account for a major part of 48–84% of MPs pollutants. Sources and abundance of MPs in Qinghai Lake, the largest inland lake in China, were investigated by Wu et al.^[^ ^4^ ^]^ In this study, the abundance of MPs in surface water and inflowing rivers were up to 7.58 × 10^5^ items km^−2^ and 0.31 × 10^5^ items km^−2^, respectively. It was also identified that PE and PP are the main chemical component of these MPs. He et al. indicated that the average abundance of MPs in shallow and deep soils in the suburbs of Shanghai were 78.00 and 62.50 items kg^−1^ respectively.^[^ ^3^ ^]^ It was also indicated that the fibers (53.33%) and fragment (37.58%) were the dominant morphologies of MPs. Zhang et al. investigated the abundance of MPs in sediment samples from the Qiantang River and Hangzhou Bay, in which the microplastic abundance of 0.23 and 0.18 particles g_sediment_^−1^ were detected, respectively.^[^ ^44^ ^]^ Micro‐Fourier transform infrared spectroscopy analysis identified that PE‐MPs accounted for 60% of the total items of MPs. Owing to the low density and small size of MPs, wind, precipitation, surface runoff, and riverine transport result in the worldwide migration of MPs. As the final destination of all kinds of pollutants from the land, ≈15–51 trillion MPs, equivalent to 93 000–236 000 tons in weight, have been accumulated in the ocean since 2015.^[^ ^45^ ^]^ As previously projected under the current production and waste of plastics, the total amount of plastic wastes and MPs accumulated in ocean could increase by an order of magnitude from 2010 levels by 2025.^[^ ^15^ ^]^ Moreover, Kane et al. found a high MPs abundance of up to 1.9 × 10^7^ items m^−2^ in “hot spots” created by ocean current.^[^ ^46^ ^]^ In addition, the presence of MPs has been found at both the proximity of human habitats and remote areas far away from human activities. MPs pollution is also a serious problem in Mariana Trench, where the MPs abundance was reported to be about 2200 pieces L^−1^ (6.20 pieces g_sediment_^−1^) at depths of 5108–10 908 m.^[^ ^47^ ^]^ MPs pollution also exists in drinking water. Based on the statistics reported by Craver et al., the concentrations of MPs with particle size between 1 and 500 µm in drinking water could be as high as 5505 items L^−1^.^[^ ^23^ ^]^

Although ingestion of MPs does not directly lead to acute fatal effects on organisms, chronic toxicity could be triggered by long‐term exposure to MPs.^[^ ^48^ ^]^ Sussarellu et al. pointed out that the intake and transfer of polystyrene (PS) MPs in blood can cause reproductive disruption in marine filter feeders.^[^ ^49^ ^]^ It should be noted that the shape, size, surface charge, dose, and other factors of MPs play a critical role on inducing biological toxicity.^[^ ^50^ ^]^ Au et al. reported that fibers PP‐MPs were more toxic than PE‐MPs particles on significantly declining of the food egestion of *Hyalella azteca*.^[^ ^51^ ^]^ Zhang et al. proved that intestinal inflammation was easily induced by the activation of *TLR4* signaling when exposed at a high‐concentration of PE‐based MPs.^[^ ^52^ ^]^ During the photodegradation and biodegradation of MPs in environment, different functional groups (e.g., —COOH and —NH_2_) can be introduced onto their surfaces and thus alter their surface charge. Kim et al. found that the PS NPs could be internalized by alveolar cells, and the positively charged NPs could result in the activation of apoptotic signaling.^[^ ^38^ ^]^ To give the as‐manufactured plastics certain application, various additives such as low molecular organics, polymers, and heavy metal complexes are used as plasticizers, flame retardants and colorants and incorporated into plastics. Unfortunately, most of the additives such as, bisphenol A, di‐2‐ethylhexyl phthalate, and heavy metal complexes are known to be toxic.^[^ ^53^, ^54^ ^]^ Thus, the additives leaching from MPs can also impose toxicity to biota and human. As previously mentioned, the relatively high specific surface area and small particle size of the MPs resulted in the adherence and accumulation of heavy metals^[^ ^16^ ^]^ and POPs^[^ ^17^, ^18^ ^]^ on the surface of MPs, which then further migrate in the environment with MPs. Toxic elements such as, cadmium (Cd), lead (Pb), mercury (Hg), and arsenic (As), as well as, polycyclic aromatic hydrocarbon (PAHs) and polychlorinated biphenyls (PCBs) have been detected on the surface of MPs samples obtained from various environments.^[^ ^25^ ^]^ Turner et al. showed that the concentrations of Pb encountered in MPs was up to 17 500 µg g_MPs_^−1^ in the sediment of beaches in southwest England.^[^ ^16^ ^]^ PAHs and PCBs carried by MPs were detected with remarkably high concentrations of 13 708 and 7554 ng g_MPs_^−1^ by Taniguchi et al. in the coast of the state of São Paulo.^[^ ^55^ ^]^ In addition, MP particles can also act as carriers of foreign species and thus transporting the potentially pathogenic microorganisms in the environment.^[^ ^56^, ^57^, ^58^ ^]^ Due to the accumulation effect, concentrations of these hazardous matters can be 1 million times higher than those in the ambient environment,^[^ ^19^ ^]^ which results in the enhanced toxicity triggered by the ingestion of MPs.^[^ ^59^, ^60^, ^61^ ^]^ Hence, it is highly essential to develop efficient solutions for tackling the MPs issue.

## 3 Current Management Approaches of Microplastics

Due to the heavy use of plastic microbeads as additives in personal care products and the release of microfibers during laundry of synthesized textiles, municipal wastewater contains a large number of MPs. Before entering the environment, waste water is treated in WWTPs. Although WWTPs are regarded as reliable filters to trap all kinds of pollutants existing in the municipal water, it has been reported that the effluent from WWTPs is actually a key outlet for releasing MPs to our natural environment.^[^ ^19^, ^23^, ^40^ ^]^ Due to the lack of effective MPs removal technologies in WWTPs, numerous MPs can pass through traditional WWTPs and enter the natural water systems. Moreover, MPs that are removed by waste water treatment are mainly retained in the sludge, which is mostly directly landfilled or further processed as farmland fertilizer. These MPs can still return to water systems through soil erosion or surface runoff.^[^ ^26^ ^]^ Due to the comprehensive natural forces of wind, rainfall, and snowfall, MPs now widely existing globally.^[^ ^62^, ^63^ ^]^ Mechanical collection of the MPs already existing in the environment by trawl or automatic collectors is helpful to purify the natural water bodies, yet is highly time‐consuming and is far away from being efficient and effective. Rational regulation of MPs in WWTPs is therefore of great importance to block MPs from further migration into the environment.

### 3.1 Water Waste Treatment Plants and Microplastic Removal Efficiency of Each Step

**Figure 3:**
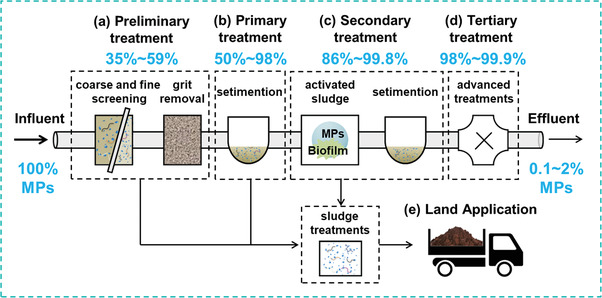
Removal of MPs at different WWTP steps: a) Preliminary treatment. b) Primary treatment. c) Secondary treatment. d) Tertiary treatment. e) Land application.

A typical waste water treatment process in WWTPs comprises four stages: Preliminary treatment, primary treatment, secondary treatment, and tertiary treatment (**Figure** **3**).

The first step of WWTPs is a so‐called “pre‐treatment” process, comprising a preliminary and a primary treatment step (Figure 3a). At the first stage of the entire process, rags, sticks, and other large items are trapped during the preliminary treatment process to avoid their damage to pumps and interference of membranes in the subsequent purification processes. It typically comprises coarse screening (6–150 mm), fine screening (less than 6 mm), and grit removal steps. Large flocs of fat, oil, and grease contained in wastewater can be helpful to trapping MPs during preliminary treatment.^[^ ^64^ ^]^ Thus, the suspended or floating MPs are removed along with other insoluble impurities affording an ≈35–59% MPs removal efficiency at this stage.^[^ ^19^, ^65^ ^]^ Subsequently, a portion of the remaining MPs are removed by gravity separation and surface skimming operations in primary clarifiers (Figure 3b), where the relatively heavier MPs can settle down or be trapped in sludge flocs, while the floating lighter MPs are trapped by the grease during surface skimming. As a result, about 50–98% of the MPs can normally be removed after primary treatment.^[^ ^66^ ^]^ During the pre‐treatment process, the removal efficiency of MPs is influenced by several characteristics of the MPs, including their size, chemical compositions and morphology. Dris et al. indicated that the concentration of the relatively larger MPs with particle size of 1–5 mm was significantly reduced from 45% in the influent to 7% in the effluent during pre‐treatment process.^[^ ^67^ ^]^ Murphy et al. found that the lighter PE‐MPs floating on the water surface can be easily removed in the skimming process, while the heavier polyvinyl chloride (PVC) and polyethylene terephthalate (PET) MPs can be separated by settling or being captured by sludge flocs.^[^ ^40^ ^]^ It is worth noting that the concentration of fibrous MPs in waste water are markedly reduced in the pre‐treatment effluent, which could be ascribed to the long‐shaped fibrous MPs are more prone to be removed by coagulation trapping and following gravity separation.^[^ ^68^ ^]^ However, it has been reported that fibrous MPs remain to be the largest fraction after this stage.^[^ ^64^, ^69^ ^]^ In addition to the retention by pre‐treatment, the decrease of particle size of MPs in effluent could be ascribed to the fragmentation of large items by the physical forces through sand abrasion or water turbulence.^[^ ^70^ ^]^ This phenomenon leads to the newly generated smaller sized MPs debris passing through pre‐treatment and proceeding to secondary treatment.

The next step of WWTPs is the secondary treatment, where the remaining suspended solids and dissolved organic pollutants in the water can be further removed by the combined use of the activated sludge and the clarification tank (Figure 3c). Aside from being captured by sludge, the microorganisms in the activated sludge can enhance the MPs removal efficiency. Due to growth of biofilms on the surface of MPs, the relative densities of MPs are normally dramatically altered, which facilitates the sinking and subsequent separation of suspended MPs.^[^ ^71^, ^72^ ^]^ It is known that there are a few bacteria, such as, *Bacillus*, *Rhodococcus*, and *Nocardia asteroids*, that are capable of degrading MPs,^[^ ^73^, ^74^, ^75^ ^]^ however, the low biodegradation rates and short contact times between activated sludge and MPs result in negligible effect on the degradation of MPs in secondary treatment. The overall MPs removal efficiency is 86–99.8% relative to the pre‐treatment effluent accompanied with decrease of the MPs’ average size.^[^ ^71^, ^72^ ^]^ Sun et al. reported the MPs with diameters larger than 500 µm were completely removed in this stage and the remaining ones were predominantly smaller than 190 µm.^[^ ^19^ ^]^ Talvitie et al. revealed that only 8% MPs in the secondary effluent are larger than 300 µm in diameters.^[^ ^76^ ^]^ Similar to the pre‐treatment stage, MPs undergo the continuous fragmentation under the comprehensive physical and biological factors. Although the formation of biofilms on the surface of MPs can facilitate the sedimentation of the MPs, the altered surface wetting properties and their relative densities might lead to MPs escape from skimming and settling processes.^[^ ^19^ ^]^ For many WWTPs, the secondary effluent is discharged into water environment after disinfection, causing the leakage of large amounts of fibrous MPs and smaller‐sized (i.e., <300 um) MPs which accumulate in the environment. As indicated in a report, a high MPs removal efficiency of 98% was achieved for a secondary WWTP serving 650 000 people in Scotland. However, the daily seepage amount of MPs (corresponding to the 2% remaining) into the environment was estimated to be 65 million pieces.^[^ ^40^ ^]^ In a separate report, the estimated median value of the amount of MPs discharged from WWTPs with an average annual efflux of 5 × 10^7^ m^3^ per year was 200 000 particles per day.^[^ ^77^ ^]^

Tertiary treatment, also called as advanced treatment, is the final stage of the whole process, which is frequently adopted to produce high quality drinking water (Figure 3d). Typically, gravity filtration, sand filtration, disc filters, dissolved air flotation, biologically active filters, membrane bioreactors (MBRs), and other advanced treatments can be applied to decrease the concentration of suspended solid impurities, organic pollutants, heavy metals, and pathogens in water.^[^ ^69^ ^]^ Based on these separation techniques, it has been reported that a high MP removal efficiency of 98–99.9% can be delivered. It is interesting to note that the fibrous MPs are difficult to remove in this process. By employing a sampling device with multiple mesh screens (500, 190, and 100 µm) to collect MPs from tertiary effluent, Ziajahromi et al. found that the MPs smaller than 190 µm are the majority of the residual MPs in tertiary effluent.^[^ ^78^ ^]^ Meanwhile, the fibrous MPs have been proved to be the major species (54.5–88.9%) in tertiary effluent, which is thought to arise as these high‐aspect‐ratio materials are able to pass through the pores of filter or membranes longitudinally.^[^ ^39^, ^65^, ^69^, ^78^ ^]^

Despite the relative high MPs removal efficiency in WWTPs, the remaining MPs are extremely difficult to be removed by using currently available technology. Development of more advanced techniques to reduce the amount of MPs in the effluent of WWTPs is urgent to avoid their further migration into the environment. In the next sections, we will discuss the recent progresses to regulate MP based on several advanced techniques.

### 3.2 Physical Trapping Methods

#### 3.2.1 Coagulation

In current water plants, coagulation is frequently used during the advanced treatment to produce drinking water of high quality. The coagulants, for example, ferric sulfate or aluminum sulfate, used in this procedure triggers the aggregation of the suspended particulate matters into flocs^[^ ^19^, ^65^ ^]^ which subsequently sediment and can be conveniently separated from water. The hydrogen bonding and/or electrostatic interactions between the coagulants and suspended solids are the key to ensure good separation efficiency during this process.

To investigate the efficacy of MP removal via coagulation, Ma et al. studied a Fe‐based salt (FeCl_3_·6H_2_O) as coagulant to remove PE MPs at pH 7.0 in a lab‐scale simulated drinking water treatment.^[^ ^79^ ^]^ This process was not very efficient with only 13% MPs with diameters <0.5 mm able to be removed at a relative high Fe salt concentration of 2 mmol L^−1^ (≈112 mg L^−1^ Fe), where the doses of Fe commonly applied for coagulation of other impurities in drinking water treatment were ≈20 mg L^−1^. The authors believed that some MPs trapped in unstable flocs were not efficiently settled, which could be ascribed to the weak interaction between the coagulant and the surface of pristine plastic.^[^ ^71^ ^]^ By simply adding 15 mg L^−1^ polyacrylamide (PAM) to the FeCl_3_.6H_2_O, an excellent MP removal efficiency of 90.91% ± 1.01% was achieved. The authors speculated that, due to static interactions between the anionic PAM and cationic Fe‐based flocs, more stable and dense flocs were formed and the escape of MPs from them was therefore effectively avoided.

In addition to the use of additives, the surface properties of MPs is a significant aspect influencing the MPs removal efficiency of coagulation.^[^ ^80^ ^]^ For instance, the changes of surface chemistry and roughness of MPs during the weathering processes in the natural environment could impact MPs affinity for coagulants and flocculants. Lapointe et al. evaluated the coagulation efficiencies in removing pristine and weathered MPs.^[^ ^81^ ^]^ In this study, PE and PS microspheres and polyester microfibers were the main subjects during the aluminum salts‐based coagulation (alum concentration of 0.063 mmol L^−1^). For the pristine MPs removal, a superior removal efficiency of polyester microfibers of 99% and residual MPs of 5 microfibers L^−1^ were delivered, while the removal efficiencies of PE and PS microspheres was 82% (residual of 90 MPs L^−1^) and 84% (residual of 80 MPs L^−1^), respectively. This phenomenon can be not only attributed to the stronger adsorption bridging effects between the fibrous polyester MPs and flocs, but also ascribed to the enhanced surface attachment of flocs resulting from the presence of abundant C = O bonds at the ester group. For the weathered MPs in environment, the photo‐oxidation effect and the attachment of natural organic matter (biofilm or POPs) can introduce oxygen‐containing groups (hydroxyl and carboxylic acid groups) and unsaturated bonds (vinyl groups). Thus, these newly formed groups could promote interactions between the coagulant and MPs by acting as anchoring sites. As a consequence, the removal of weathered PE was remarkably increased to 99%, compared to the 82% of pristine PE.

In summary, the development of advanced and green additives to stabilize the flocs, as well as, the extra oxidation steps (e.g., UV and ozone) to boost the interaction between flocs and MPs are critical to enhance the MPs removal efficiency during coagulation process.

#### 3.2.2 Membrane‐Based Filtration

Due to key advantages of a high separation efficiency and compact plant size, filtration techniques such as, microfiltration (MF), ultrafiltration (UF), reverse osmosis (RO), dynamic membranes (DM), and MBRs, have been shown to be feasible to produce high quality water from primary or secondary effluent.^[^ ^82^, ^83^ ^]^ By using the asymmetric membranes with micrometer or nanometer‐sized pores, the impurities including bacteria, protozoa, viruses, and the suspended solids can be effectively removed. Although UF and RO have exhibited the good efficiency for MPs removal in WWTPs, millions of MPs still remained in the effluent after these treatments.^[^ ^78^ ^]^

Dynamic membrane technology has been studied extensively as a promising candidate in advanced water treatment process.^[^ ^84^, ^85^ ^]^ Different from traditional filtration approaches that use membranes with micrometer or nanometer‐sized pores to retain the tiny solids, DM technology uses a newly formed cake layer on supporting membrane through filtration, which serves as a secondary barrier to trap the impurities debris. Since the DM is fully composed of the solid impurities from the wastewater, the use of extra chemical agents can be avoided and the possibility of introducing secondary pollutants can be therefore circumvented. Typically, the low filtration resistance and low trans‐membrane pressure (TMP) during DM filtration make it feasible to be applied under a gravity‐driven mode without the use of pumps. By utilizing a gravity‐driven lab‐scale DM filtration protocol, Xu et al. attempted to purify synthetic wastewater composed of tap water and diatomite particles.^[^ ^86^ ^]^ The pore size of the supporting membrane is 90 µm, while the sizes of 90% of the diatomite particles are in a range of 1–90 µm. After 20 min of treatment, the turbidity of the synthetic wastewater was significantly reduced from 195 to 1 nephelometric turbidity unit, indicating a high removal efficiency of these micro‐sized MPs by DM technology. In addition, the TMP during DM filtration is significantly lower than UF and OR, resulting in overall lower energy consumption.

Recently, membrane technologies have been further modified by coupling with other techniques, overall exhibiting higher MPs removal efficiency. MBR, a heterogeneous reaction system composed of a biological reactor and a membrane system, has been developed for advanced wastewater treatment.^[^ ^87^ ^]^ First, the influent enters the bioreactor and undergoes a biodegradation process. Here, biological‐activated sludge with high biomass concentration and relative long solid retention time allows for an efficient removal of refractory organic pollutants. Subsequently, a semi‐crossflow filtration system is utilized to separate the mixed suspension, where the suspend solids are intercepted by membrane and concentrated in the retentate. With the synergy of the bio‐reaction processes and porous membranes, the MBR exhibited superior performance for treatment of MPs in contaminated wastewater. Talvitie et al. reported a MBR set‐up by integrating a biodegradation tank with a membrane filtration tank composed of 20 submerged flat‐sheet membrane units with effective membrane area of 8 m^2^ and pore size of 0.4 µm.^[^ ^88^ ^]^ By applying this technique, the concentration of MPs in a primary effluent in WWTPs was reduced significantly from 6.9 to 0.005 MP particles L^−1^, indicating a remarkably high elimination rate of 99.9%. It is noteworthy that the biological reactor permitted the degradation of organic impurities adhered onto MPs, which is beneficial to enhance the accuracy of qualitative analysis of the separated MPs. Similar to the biological process in secondary treatment, it is commonly believed that the biodegradation of chemical inert MPs is limited during the MBR process. Although filtration technique is highly promising for MPs removal in tertiary treatment, membrane fouling and abrasion are the main challenges of this technique, which often lead to the decay of purification efficiency.^[^ ^64^, ^70^ ^]^

#### 3.2.3 Adsorption

**Figure 4:**
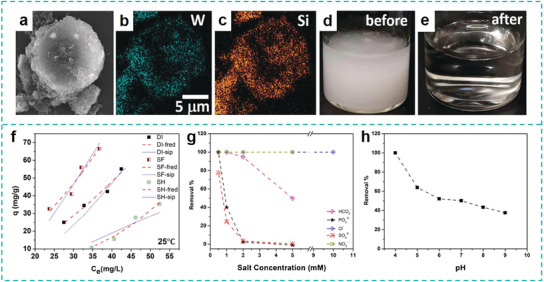
Capturing MPs through adsorption. a–c) SEM and EDX elemental mapping images of 10 µm PS beads absorbed onto the magPOM‐SILP. d,e) optical images of PS contaminated water before and after treatment. Reproduced with permission.^[^ ^90^ ^]^ Copyright 2020, Wiley‐VCH. f) Nanoplastics adsorption isotherm curves of Zn‐Al LDH at 25 °C in distilled water (DI), synthetic freshwater(SF), and synthetic hard water (SH). g) MPs removal efficiency in the presence of competitive anions. h) MPs removal efficiency under different pH. Reproduced with permission.^[^ ^91^ ^]^ Copyright 2020, Elsevier.

Adsorption has also been frequently applied to absorb pollutants such as, heavy metals and organic contaminants in water. Ion exchange, *π*–*π* interactions, hydrophobic interactions and hydrogen‐bond interactions between the absorbents and contaminates are responsible for the adsorption performance. Recently, this method has also been employed to mitigate MPs in water treatment process. Yuan et al. investigated the adsorption efficiency of PS MPs by using 3D reduced graphene oxide (3DRGO).^[^ ^89^ ^]^ In this study, PS microspheres with an average diameter of 5 µm were used as MP samples. Because of the strong *π*–*π* interaction between 3DRGO and PS‐MPs, an excellent adsorption capacity of 617.28 mg_PS_ g_3DRGO_^−1^ was obtained under the mild experimental conditions (pH 6, 26 °C). It should be noted here, it is expected a sp^2^‐rich MPs would adsorb strongly on RGO. Given sp^3^‐rich MPs such as, PP and PE dominate the total amount of MPs in the environment,^[^ ^4^ ^]^ the study would benefit from a broader focus on different polymers than do not experience *π*–*π* stacking. Streb et al. fabricated a functional nanoparticle composite by loading water‐immiscible polyoxometalate ionic liquid (POM‐IL) onto magnetic Fe_2_O_3_@microporous SiO_2_ core‐shell particles (defined as magnetic polyoxometalate‐supported ionic liquid phases, magPOM‐SILP).^[^ ^90^ ^]^ The magnetic Fe_2_O_3_ core facilitated a facile separation and recycling of absorbent. Specifically, the POM‐IL was synthesized by the combination of the lacunary‐Keggin cluster anion ([*α*‐SiW_11_O_39_]^8−^) with the tetra‐n‐heptyl ammonium cation (O_7_ ( = (n‐C_7_H_15_)_4_N^+^), where the presence of Q_7_ endows a high hydrophobicity of POM‐IL. Thus, the water‐immiscible POM‐IL allowed a fast attachment of PS beads (with diameter of 1 and 10 µm, concentration of 1 g L^−1^) on the surface of these particles due to the hydrophobic interactions between POM‐IL layer and the PS (**Figure** **4**a–c). As a result, a complete removal of PS beads in 5 mL deionized water within 24 h was quantified by dynamic light scattering (Figure 4d,e). Moreover, the MPs removal performance of larger volumes of water (50 mL) was also studied, where the absorbent delivered an over 90% MPs removal efficiency equivalent to an excellent removal capacity of 900 mg_PS_ g_magPOM‐SILP_^−1^. In addition, this novel magnetic absorbent opened up a new avenue to overcoming the challenge of absorbent separation in practical water treatment applications by circumventing the time consuming conventional filtration.

Although MPs attract wide spread attention, nano‐sized plastics, NPs, also widely exist. NPs have a higher removal resistance in current water treatment processes and are even more hazardous to the ecosystem. To enhance the adsorbing efficiency of NPs, Darbha et al. designed a type of double‐layered Zn‐Al hydroxide (Zn‐Al LDH) to adsorb PS particles with diameters smaller than 1 µm from aqueous solution.^[^ ^91^ ^]^ The surface properties of MPs existing in the natural environment are dramatically altered to be more negatively charged due to oxidation. Thus to mimic this effect, PS NPs were pretreated by coating with anionic surfactant.^[^ ^25^ ^]^ This system is characterized by a superior anionic exchange capacity (AEC ≈ 3 meq g^−1^) and thus has strong adsorption ability for the anionic contaminants. Moreover, partial substitution of the trivalent Al ion by the divalent Zn ion affords a positively‐charged surface of LDH facilitating its sorption of anionic species. Based on the adsorption isotherm curves (Figure 4f), the maximum adsorption capacity toward PS NPs was calculated as 164.49 mg_PS_ g_Zn‐Al LDH_^−1^, 162.62 mg_PS_ g_Zn‐Al LDH_^−1^, and 53.2 mg_PS_ g_Zn‐Al LDH_^−1^ in deionized water (DI), synthetic freshwater (SF), and synthetic hard water (SH), respectively. The authors pointed out that to avoid the competitive absorptions and reduction of adsorption capacity by anions such as HCO_3_^−^, PO_4_^3−^, and SO_4_^2−^, these ions in water should be removed in prior to the addition of Zn‐Al LDH (Figure 4g). In addition, the performance of this adsorbent under basic condition was unsatisfactory, which is related to the decreased zeta potential of Zn‐Al LDH resulting from deprotonation of hydroxyl groups (Figure 4h). Therefore, it is challenging to directly apply this system in a complex water system with varied unknown anions.

In addition to research works mentioned above, various absorbents such as, Fe_3_O_4_‐CNT and Zr‐MOFs‐based foam have been developed and delivered superior performance in other typical MPs (i.e., PE, PET, PMMA, PVDF, and PA) removal.^[^ ^92^, ^93^ ^]^

In general, MPs removed during the multi‐stage wastewater treatment processes remain in the sludge. This MPs rich sludge is often further processed either as landfilled or used as farmland fertilizer (Figure 3e).^[^ ^94^ ^]^ Although a series of treatments are applied to remove harmful matter before the application in agriculture, the presence of MPs is ignored.^[^ ^95^ ^]^ Thus, unfortunately, no matter how many developments have been done, these strategies mentioned above based on simple physical separation cannot permanently address the pollution of environmental MPs, as the lack of rational treatment to recycle or completely eliminate the separated and collected MPs debris and particles.

### 3.3 Chemical Degradation Approaches

This re‐introduction of physically separated MPs into the environment has led to the development of chemical strategies to degrade, and thus permanently remove MPs. In this process, catalysts play significant roles to generate reactive oxygen species (ROSs) and thus trigger the degradation of MPs.

#### 3.3.1 Advanced Oxidation Processes

AOPs are known as a powerful method to eliminate organic pollutants through generating ROSs with high standard reduction potentials, such as, sulfate radical (SO_4_^•−^, *E*_0_ = 3.1 V vs NHE) and hydroxyl radical (^•^OH, *E*_0_ = 2.7 V vs NHE).^[^ ^96^, ^97^, ^98^ ^]^ Due to their strong oxidation capability, a large variety of pollutants including dyes, antibiotics, and POPs have been effectively degraded or mineralized by this technique.^[^ ^99^ ^]^ As a unique type of organic pollutant, MPs are obviously more challenging to be degraded due to their considerably higher molecular weights (MWs) compared to other low MW organic pollutants as mentioned above. A few pioneer works reported recently are ambitious to degrade MPs via this protocol. Though previous works have proved the ROSs generated via AOPs could destroy the surface structure of MPs particles,^[^ ^100^ ^]^ the investigations of AOPs in MPs pollutants degradation are extremely rare.

**Figure 5:**
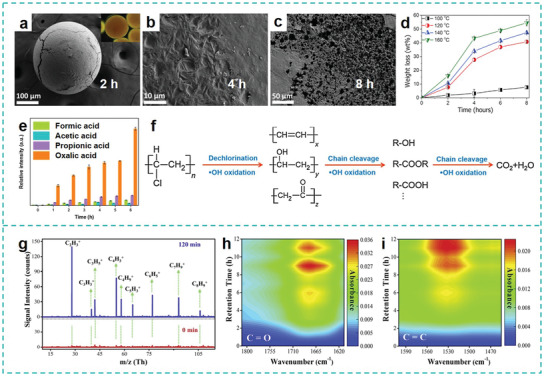
AOPs and photocatalytic for MPs degradation. a–c) SEM images of MPs after 2, 4, and 8 h of degradation. d) Weight loss of MPs under different temperature. Reproduced with permission.^[^ ^101^ ^]^ Copyright 2019, Elsevier. e) Evolution of organic intermediates during the electro‐fenton treatments. f) Mechanism of degradation of PVC. Reproduced with permission.^[^ ^108^ ^]^ Copyright 2020, Elsevier. g) Mass spectra obtained by HPPI‐TOFMS during the photodegradation of PS. DRIFTS study of PS at different time intervals: h) C = O signal, i) C = C signal. Reproduced with permission.^[^ ^112^ ^]^ Copyright 2020, Elsevier.

Wang et al. were the first to use SO_4_^•−^‐based AOPs (SR‐AOPs) to degrade MPs. In their report, a helical‐shaped, N‐doped carbon nanotube catalyst encapsulated with manganese carbide nanoparticles (Mn@NCNTs) was fabricated for SR‐AOPs reaction, while PE beads (Figure 7b) obtained from a few commercial facial cleansers were used as MP samples.^[^ ^101^ ^]^ Under a hydrothermal (HT) condition created in an autoclave, the PE‐MPs beads first generated cracks, fused into a thin polymer film and then progressively developed into a film with a large amount of cavities as evidenced by SEM analysis (**Figure** **5**a–c). A remarkable 54 wt% weight loss of MPs was achieved by this method after reaction for 8 h at 160 °C (Figure 5d). The decay of MPs removal efficiency in the first three cycles was slight, indicating the outstanding stability of Mn@NCNTs in the SR‐AOPs system. From the perspective of the reaction mechanism, the authors verified that the presence of SR‐AOPs is indispensable, which continuously supplies the free radicals (SO_4_^•−^ and ^•^OH) to oxidize MPs particles into small molecules and then mineralizing them into CO_2_ and H_2_O. A hydrothermal condition plays a crucial role in this process as well: The generated bubbles and vapor mechanically shear the PE‐beads causing chain scission of the macromolecules and triggering the degradation process; meanwhile, the generation of ROSs is also effectively accelerated given that the activation of peroxymonosulfate (PMS) can be heat‐driven. The authors proposed that the mechanism of this process is as such: The C—C bond of PE backbone is first broken into two hydrocarbon radicals under a HT conditions. Subsequently, when further decomposition of the hydrocarbon radicals occurs, the lower‐weight molecules are generated and are further converted into new shorter‐chain hydrocarbon radicals via the *β*‐scission and hydrogen abstraction routes, induced by other hydrocarbons. Finally, these intermediates radicals are attacked and ultimately mineralized by the SO_4_^•−^ and ^•^OH generated from SR‐AOPs system. In this manner, the highly persistent PE was degraded into environmentally‐benign intermediates such as low hydrocarbon length aldehyde, ketone, and carboxylic acids, which were biodegradable and could be utilized as carbon sources for algae growth. As the SR‐AOPs reaction progress, these intermediates were degraded into low molecular weight organics and further mineralized into CO_2_ and H_2_O. Although the relatively harsh reaction conditions (high pressure and high temperature) is not feasible to be directly applied in the WWTPs for MPs removal, this interesting piece of work provides a good example to demonstrate to which extent the chemical degradation process is strengthened to break down the highly persistent C‐C bond based macromolecules.

Alternatively, EAOPs based on Fenton or Fenton‐like chemistry (electro‐Fenton, EF) are most frequently utilized to generate ROSs (such as ^•^OH) and decontaminate the persistence organic pollutants (POPs), which are featured as versatility, superior efficiency, excellent environmental compatibility and sustainability.^[^ ^102^ ^]^ During this process, the cathode material plays the key role in determining the degradation efficiency of the system: Hydrogen peroxide (H_2_O_2_) is generated in situ on the cathodes by reducing O_2_ via a two‐electron oxygen reduction reaction and is subsequently converted into ^•^OH.^[^ ^103^, ^104^, ^105^, ^106^, ^107^ ^]^ Gao et al. designed a heterogeneous TiO_2_/C cathode in a EF‐like system for degrading PVC‐based MPs in a Na_2_SO_4_ electrolyte (0.05 m, pH 3.0), where a graphite electrode and Ag/AgCl electrode were used as the counter electrode and reference electrode, respectively.^[^ ^108^ ^]^ At the end of the reaction, the original smooth surface of PVC‐MPs was destroyed by showing a number of large holes according to SEM characterization. After potentiostatic electrolysis at −0.7 V under 100 °C for 6 h, a dechlorination and degradation efficiency of 75% and 56 wt% were obtained, respectively, through quantitative analysis of the concentration of Cl^−^ in the electrolyte and weight loss of the PVC MPs. The evolution of organic intermediates (e.g., formic acid, acetic acid, propionic acid, oxalic acid) during the EF treatments further proved the destruction of PVC into small organics (Figure 5e). The input of electrons in such EF‐like systems continuously boosts the generation of catalytic species. By characterizing the chemical structures of degrading intermediates and residual material in the electrolyte, the authors proposed the reaction mechanism of dechlorination and degradation process as depicted in Figure 5f. First, the direct electrons transfer from TiO_2_/C cathode to the PVC MPs gives rise to the dechlorination under heating conditions. Meanwhile, the attack of oxidative ^•^OH toward PVC MPs results in the formation of oxygen‐containing groups such as C = O and O—H, combined with the release of liquid shorter‐chain intermediates. Subsequently, the vulnerable hydrocarbon species are prone to be oxidized into small molecular weight organics and finally mineralized to CO_2_ and H_2_O. Given the advantages of the relative mild conditions under 100 °C and −0.7 V (vs Ag/AgCl), such an EF‐like technology is promising for potential applications in WWTPs to purify the MPs‐contaminated water bodies.

#### 3.3.2 Photocatalysis

Photocatalysis is a well‐established environmentally‐friendly technique exploiting free and inexhaustible solar energy. In recent years, this technique has gained significant applications in water purification because of its high degradation efficiency of antibiotics, pesticides, and dyes.^[^ ^109^ ^]^ The mechanism of photocatalytic degradation process can be attributed to the interaction between ROSs (e.g., hydroxyl (^•^OH), superoxide (O_2_^•−^)) generated on the surface of semiconductors and the organic substrate, which breaks the chemical bonds of organic pollutants leading to their complete mineralization toward CO_2_ and H_2_O. Meanwhile, the photo‐excited holes (h^+^_VB_), generated by transfer of electrons from the valance band to the conduction band, are capable to directly oxidize the organics into CO_2_ and H_2_O.

TiO_2_ is a classical photo‐catalyst capable of oxidizing organic contaminants with high efficiency. It also has several advantages, including low toxicity, cheap price and excellent acid and alkali‐resistance.^[^ ^110^, ^111^ ^]^ In recent years, the TiO_2_‐based materials have been extensively studied in photocatalytic degradation of MPs. Due to a relatively high band gap of 3.2 eV, a TiO_2_‐catalzyed photo reaction is often performed under UV irradiation. Zhang et al. synthesized a TiO_2_ film as catalyst to degrade PS microspheres and PE powder at solid‐state under UV irradiation.^[^ ^112^ ^]^ As the essential part of the photocatalytic system, the TiO_2_ films were fabricated by dropping TiO_2_ nanoparticles (P25) dispersed in a mixture of water, ethanol and Triton X‐100 (TXT) on the conduction side of FTO glass and further calcined at 450 °C in air. The PS spheres solution with diameter of 400 nm was dropped on the TiO_2_ films and was subsequently dried for photodegradation experiment. During the annealing process, TXT served as the morphology‐directing agent to adjust the surface properties, electrical properties and porous sizes distribution. Therefore, the Triton X‐100 based TiO_2_ (TiO_2_‐TXT) film delivered superior hydrophilicity, specific surface area as well as the separation and transfer of photogenerated electron‐hole pairs. Under UV irradiation with wavelength of 365 nm, continuous fragmentation of original structures was observed and an almost complete mineralization (98.40%) of 400 nm‐PS spheres was achieved by TiO_2_‐TXT catalyst within 12 h, proving the effectiveness of this protocol. The excellent mineralization efficiency of TiO_2_‐TXT film was mainly ascribed to two aspects: 1) The unique surface hydrophilicity could improve the interaction between PS MPs and TiO_2_; 2) the enhanced charge carrier generation and separation could accelerate the generation of ^•^OH and O_2_^•−^, which act as the significant role in the degradation process. In addition to the oxidation degradation triggered by ROSs, photo‐excited holes also contributed toward oxidizing and mineralizing MPs into CO_2_ and H_2_O. Further measurements by high‐pressure photon ionization‐time of flight mass spectrometry and in situ diffuse reflectance infrared Fourier transform spectroscopy revealed that PS were degraded into multiple intermediates including styrene, benzene, toluene (Figure 5g) based on the presence of their corresponding characteristic signals of functional group (i.e., C = O and C = C) (Figure 5h,i). Meanwhile, the degradation of PE also presented marked change in morphology and chemical bonds, suggesting the feasibility and potential of this method in degrading various MPs contaminations.

To fill the knowledge gap in the underlying effect of operating parameters such as pH and temperature during degradation process, González et al. investigated the degradation behaviors of HDPE primary MPs with the presence of a newly designed C, N‐doped TiO_2_ catalyst,^[^ ^113^ ^]^ which absorb light with wavelength of 428 nm, within the range of visible light. All the experiments were performed by adding HDPE and photocatalyst in water inside a batch‐type container placed in a closed reaction chamber equipped with a LED lamp emitting visible light. Optical images illustrated the different destroyed morphologies of the PE microbeads under various pH value and temperature. Under 0 °C and pH 3, a maximum mass loss of ≈72% was delivered within 50 h. Thus, the authors presumed that a synergetic effect of the low temperature and high H^+^ ion concentration exist on PE MPs photocatalytic degradation. On one hand, it is prone to form more hydroperoxide (—CH_2_—HCOOH—CH_2_‐)*_n_* at low pH, where the high concentration of H^+^ ions could serve as the promoter to boost the relevant reaction. Subsequently, two new free oxy and hydroxyl radicals ((—CH_2_—HCO^•^—CH_2_—)*_n_* and ^•^OH, respectively) were formed via the splitting of hydroperoxide, since the O−O bond was vulnerable to cleavage. These hyperactive radicals efficiently abstract protons from other polymer chains, playing a crucial role for PE degradation. Moreover, the high zeta potential of TiO_2_ nanoparticles at low pH could prevent their agglomeration and result in a good dispersion of the catalyst; the strong interaction between the well‐dispersed colloidal nanoparticles and MPs guaranteed efficient photodegradation. On the other hand, the low temperature was also favored for the degradation process. Smaller MPs particles could be formed by the fragmentation of MPs beads at 0 °C, which allowed the fast degradation through the enlarged interface between the MPs and semiconductors.

### 3.4 Overview of Current Microplastics Removal Strategies

**Figure 6:**
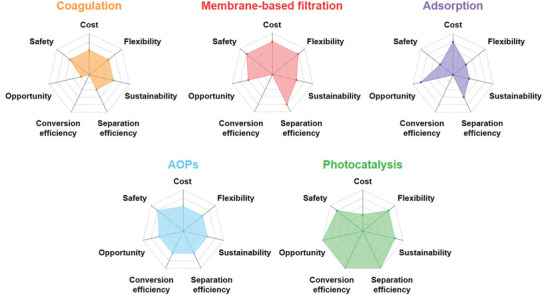
Comparison of the general performance metrics for current MPs removal strategies.

With so many potential avenues for MP removal, accurately assessing the strengths and weaknesses is critical for each approach (**Figure** **6**). Particularly, these approaches have been compared from seven aspects: Cost, safety, flexibility, sustainability, separation efficiency, conversion efficiency, and opportunity. Cost depends on the fabrication of the key materials (i.e., membrane, absorbent, and catalyst) and energy consumption during the whole process. Safety hinges on whether the toxic chemical agents are used and toxic intermediate products are generated. Flexibility: The stability of performance in practical application under various environment factors including pH, temperature, and the presence of distractions such as, other anions and organic pollutants. Sustainability is estimated from two aspects: 1) The structure and performance stability and recyclability of the key materials in the long‐term application; 2) the treatment time and experimental condition (i.e., high temperature, high press, electricity, and UV‐light). To highlight the difference between physical trapping and chemical degradation, the removal efficiency is further distinguished as separation efficiency and conversion efficiency. Chemical degradation approaches can not only separate MPs from water body but also convert them into non‐toxic matters, while MPs collected by physical trapping methods are likely to return to the environment. Overall, opportunity is the summary and outlook of these approaches in practical application.

Although coagulation and adsorption can deliver good capacity (≈90% efficiency) for MPs separation, the regeneration of coagulants and adsorbents will lead to the release of MPs into environment. Meanwhile, no one can guarantee that the leakage of organic coagulating additives and micrometer sized adsorbents will not seriously threaten the ecosystem. As the most reliable methods, membrane‐based filtration can exhibit higher efficiency than coagulation and adsorption. The flexibility of membrane‐based filtration makes it able to be coupled with other techniques and deliver enhanced efficiency. For instance, an amazing MPs removal efficiency of 99.9% could be delivered by the MBR. For the practical applications, the management and maintenance should be improved to avoid the membrane fouling and abrasion, which is essential to guarantee the sustainable purification efficiency. Although these traditional technologies are relatively developed and have delivered good MPs removal efficiencies, they can only separate the MPs from water system rather than eliminate the contaminants, which cannot completely solve the MPs issue.

As with the mitigation strategies for other persistent pollutants or greenhouse gases, chemical catalytic conversion is the most promising strategy for permanent removal of contaminants, either via elimination or upcycling into valuable products.^[^ ^114^, ^115^, ^116^ ^]^ The developments of chemical degradation of MPs through AOPs and photocatalytic degradation have given the promising alternatives of traditional technologies for thorough eradication of MPs by converting them into non‐toxic matter. Meanwhile, the development of physical‐chemical coupling approaches can be promising to further improve MPs removal efficiency by evoking a synergistic effect. More efforts should be invested to develop versatile catalysts for MPs mitigation. For instance, the incorporation of photocatalytic sites into membrane will trap MPs and deliver excellent in situ catalytic degradation performance through the shorten path of ROSs transfer and attacking. It can not only efficiently separate MPs from water but also converts MPs into non‐toxic matters rather than leaving them to accumulate and return to environment. In the near future, the combination of developed physical methods and emerging catalytic conversion approaches is essential to guarantee the overall low cost and MPs degradation efficiency in large scale water treatment applications (such as, WWTPs).

Due to rapid migration in aquatic environment, ingestion by aquatic organisms and risk to human through drinking water, MPs in water are of great concern to researchers. Previous studies always took importance on removing MPs in water, while MPs in soil were generally identified as one of the sources of MPs in water.^[^ ^1^, ^19^, ^42^, ^93^ ^]^ However, Li et al. reported MPs in soil can be also ingested by plants during growth.^[^ ^59^ ^]^ Therefore, the removal or separation of MPs from soil (especially soil in landfill with high concentration of MPs) will become an important research hot pot in the future. Commonly, density separation is the most measured for the extraction of MPs in soil, where the soil sample is added into a salt solution with defined density, and then shaken, stirred, aerated, and settled.^[^ ^1^ ^]^ Taking into account the cost and environmental effect, saturated sodium chloride (NaCl, *ρ* = 1.2 g cm^3^) solution is the most authoritative extraction medium and widely used in trace analysis and large‐scale practical applications for extracting MPs from sand or stones of soil parts (*ρ* = ≈2.6 g cm^3^). Besides, calcium chloride (CaCl_2_, *ρ* = 1.3 g cm^−3^), sodium iodide (NaI, *ρ* = 1.8 g cm^−3^), zinc chloride (ZnCl_2_, *ρ* = 1.6 g cm^−3^), and sodium polytungstate (SPT, 3Na_2_WO_4_·9WO_3_·H_2_O, 1.4–3.1 g cm^−3^) solutions have been also applied to separate certain categories of MPs. In the future development, it will be promising to design coupling strategy including density separation and the as‐mentioned physical or chemical methods for MPs remediation in aqueous systems (i.e., membrane filtration, photocatalytic degradation, and AOPs in Section 3) to achieve the separation and/or degradation of MPs in soil.

## 4 Rational Recycling Protocols of Plastic Wastes to Eliminate Microplastics

The plastic wastes in the environment are a critical issue. Numerous MPs can be released through the corrosion of mismanaged plastic wastes by sun radiation, wind and other environmental factors. Although landfill is conducted as the major management strategy for plastic wastes, the gradual release of MPs and toxic additives pose potential treats to environment. Thus, the traditional landfill is destined to be phased out in the future. Although plastic wastes could be eliminated by incineration, greenhouse gas emission is one of the most non‐ignorable impacts. The content of combustible carbon of each ton of this category of waste is ≈79%, corresponding to significant greenhouse gas emission of about 2.9 tons of CO_2_.^[^ ^117^ ^]^ Apart from the emissions of CO_2_, other harmful gases such as CO, NO, SO_2_ can be also released during open burning, accompanied by a large amount of ashes migrating and suspending in the atmosphere, bringing significant harm to the ecological environment.^[^ ^6^ ^]^ In this context, recycling is regarded as the ideal and ultimate solution for solving the current MPs issue and ensuring a sustainable use of plastics from the long run. Recycling 1 ton of plastic wastes for reuse rather than producing virgin materials can save up to ≈130 million kJ of energy.^[^ ^118^ ^]^ Moreover, recycling of plastic wastes can also effectively reduce the greenhouse gas emission. In 2014, 3.17 million tons of plastic wastes were recycled meaning that ≈3.2 million tons of CO_2_ were saved, equivalent to the gas emissions of 670 000 cars on the road in a year.^[^ ^117^ ^]^ Unfortunately, the recycle rate of plastic wastes to raw materials was low, especially for the secondary (recycled) plastics wastes.^[^ ^33^, ^119^ ^]^ The following sections describes approaches to recycle macroscale plastic wastes such that they are not naturally converted into dangerous MPs via erosion or other degradation mechanisms.

### 4.1 Typical Plastic Wastes Recycling Technologies

Typically, recycling of plastics includes four well‐known categories: Primary, secondary, tertiary, and quaternary recycling. Primary recycling is an in‐plant mechanical recycling strategy, which directly incorporates the scrap materials into prime‐grade products without any pretreatment such as, purification, physical separation, or chemical depolymerization, representing closed loop recycling. In secondary recycling, mixed plastic waste streams are sorted, fragmented, extruded and manufactured into new plastic products, which is also classified as mechanical recycling. With proper control over processing conditions, the degradation of physical properties in first several cycles can be suppressed.^[^ ^120^ ^]^ Nevertheless, the gradual thermo‐oxidative or hydrolytic scission of polymers can give rise to the decrease of quality and loss of performance, which limits the sustainability of the mechanical recycling. Several types of polymers (e.g., polyurethane) cannot be recycled mechanically. For tertiary recycling, chemical approaches are mainly involved to recover plastic wastes into oil/hydrocarbon components or high‐purity monomers by chemical bonds scissions, which can be reused as raw materials and incorporated into plastic production lines. Meanwhile, chemical recycling is a “trash to treasure” approach to upgrade plastic wastes into value‐added products. Additionally, the chemical reclamation strategy develops an extra feedstock source from disposed plastic wastes rather than the consumption of crude oil, which opens up a new avenue to relieving the crude oil crisis. Therefore, chemical recycling is considered as an attractive alternative to the traditional plastic waste management, where the “kill several birds with one stone” strategy possesses economic effectiveness and environmental friendliness. Quaternary recycling, also named as energy recovery, aims to obtain multicarbon products featured with high‐yielding heating values mainly by combustion of the plastic waste. Nevertheless, this protocol still faces several technical challenges, especially the emission of greenhouse gas and heavy metal containing ash.

**Figure 7:**
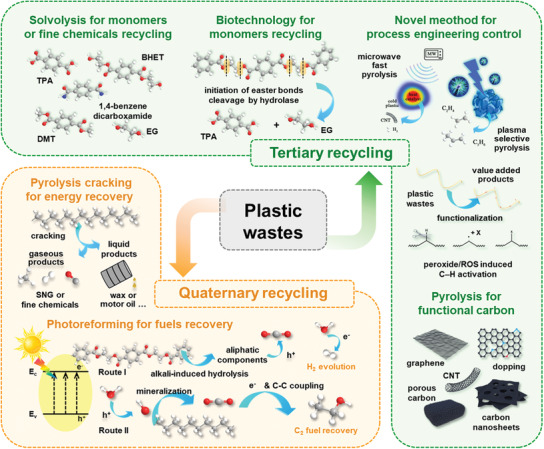
Typical plastic wastes managements using chemical approaches. Tertiary recycling: Solvolysis for monomers or fine chemicals recycling, biotechnology for monomers recycling, novel strategies for process engineering control, pyrolysis for functional carbon materials. Quaternary recycling: Pyrolysis cracking for energy recovery, photo‐reforming for fuels recovery.

Rational management of the plastic waste, especially through efficiently recycling and avoiding their leakage into the environment is undoubtedly a major pathway of cutting down the generation of the secondary MPs. In the below section, we briefly discuss the technical advances of mechanical recycling, and more detailed discussions can refer a few excellent reviews.^[^ ^121^, ^122^ ^]^ Given the high potential of obtaining high‐quality monomers or valuable chemicals from plastic wastes and achieving a close‐loop of material regeneration, as illustrated in **Figure** **7**, we will focus on the current tertiary and quaternary recycling strategies. The mechanisms, challenges, and opportunities of these techniques will be highlighted.

### 4.2 Mechanical Recycling

For mechanical recycling robust collection and separation technologies of plastic wastes are essential to obtain reasonably clean raw materials from the complex waste steams which are frequently contaminated by food, dyes, and other plastic impurities. Typically, a combination of several automated and/or manual processes are adopted to achieve this goal, including near infrared, X‐rays, density, electrostatics, melting point, hydrocyclons, selective dissolution, and manual sorting are used to enhance the efficiency of sorting.^[^ ^122^ ^]^ Recently, fluorescence marker has emerged as an advance technique to facilitate fast sorting process. Maris et al. used rare earth based compounds as fluorescent dye for plastic marking, where the marked polymers can be facilely separated under spectroscopic detection. Nevertheless, the safety of such markers have not been fully discussed/understood yet.^[^ ^123^ ^]^ Subsequently, plastics are grinded into small flakes, which further undergo sink/float methods, air elutriation, and heat discoloration for optical separation. The advantages of cheap, large‐scale, solvent‐free, and applicable to many polymers make extrusion the most frequently used mechanical recycling approach. Heat and rotating screws in the extruder lead to the thermal softening or plasticization of waste plastics, and the plastic melt further through the temperature‐controlled barrels resulted in the fabrication of fixed cross‐section extrudate. It is worth noting that free radical reactions induced thermal degradation and chain scission are accelerated by the excessive temperatures and high screw speeds, sometimes leading to the formation of downgrade or even un‐processable polymers.^[^ ^124^ ^]^ Although mechanical recycling is currently the foremost plastic recycling strategy, the recycled products are often suffered from reduced viscosity and mechanical properties, which are only applicable for low‐quality applications. Because of their lowered grade, the mechanical recycled plastics are more susceptible to environmental factors, resulting in a more rapid release of MPs into the environment.

To improve the quality of mechanical recycling, thermal, light stabilizers, and/or antioxidant are used to suppress free radical reactions during mechanical recycling. However, the presence of stabilizers complicates mechanical recycling process. For instance, carbon black is widely used as reinforcing filler and UV protector in polymers, while aesthetic requirement of the products cannot fully satisfied.^[^ ^125^ ^]^ Phenols (i.e., commercial Irganox 1010) can stabilize hydrogen bonding and trap alkyl peroxy radicals, while the migration of antioxidants and potential carcinogenicity is also a concerning issue.^[^ ^126^ ^]^ To this context, development of eco‐friendly plastic stabilizers are highly encouraged.^[^ ^127^ ^]^ A few recent examples have emerged on such aspect. For example, Iyer et al. studied the mechanical performance of LDPE extruded with a claimed low cost antioxidant derived from natural agro‐wastes,^[^ ^126^ ^]^ which effectively suppress the embrittlement of the plastics. Deeper understanding of the migration mechanism and potential toxicity of these stabilizers over several recycling life‐cycles are urgently needed.

The down‐grade of recycled plastic is characterized by the much reduced molecular weights. Therefore, the use of chain extenders to further enhance the molecular weights of the products is a cost‐effective, straightforward, and highly efficient protocol. Upon using chain extenders, the reactive end‐groups (two or more) can be conveniently jointed together by forming covalent bonds and hence significantly enhances the chain length. Oxazolines, lactams, hydroxyls, carboxylic acids, and organic phosphites and phosphates are the widely investigated the chain extenders.^[^ ^122^, ^128^ ^]^ By using triphenyl phosphite, Cavalcanti et al. successfully enhanced the viscosity of the recycled PET through their carboxyl and hydroxyl terminal groups.^[^ ^129^ ^]^ It should be noted that the potential migration and toxicity of chain extenders, where the unreacted short oligomers or other molecules may leach into packaged products. Thus, most of existing chain extenders are not suitable for food‐grade plastics production.^[^ ^122^ ^]^ More risks during the repeated extrusions could also be triggered by the limited thermal stability of chain extenders.^[^ ^130^ ^]^

Furthermore, various inorganic fillers, plasticizers, and compatibilizers have also been studied to minimize degradation during the mechanical recycling of plastics.^[^ ^131^, ^132^, ^133^ ^]^ Given the relatively short production cycle and low cost of this technique, mechanical recycling remains a key protocol in the plastic recycling system. The future development should definitely focus on a progressive enhancement of the quality and eco‐impacts of the end‐products.

### 4.3 Solvolysis of Plastic Wastes for Monomers

**Figure 8:**
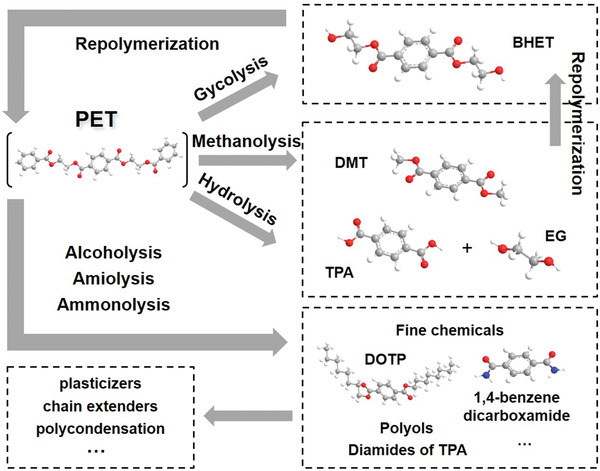
Overview of solvolysis for monomers and fine chemicals recovery of PET. Color code: C, gray; O, red; N, blue; H, White.

Monomer recycling, breaking MPs down into their own starting materials for reuse, has been regarded as one of the most efficient strategies to construct the circular economy system by potentially avoiding further exploration of the fossil feedstock and also ensuring high quality of the regenerated plastics. Various chemical depolymerization approaches for monomer recovery have been investigated and utilized thus far. Solvolysis is known as the most well‐established solution to yield high purity monomers for polymerization or other fine chemicals and reused for industrial manufacture. Since the ester bonds in polyesters, mostly referring to PET, are prone to depolymerize by a number of nucleophilic reagents, such as, water, alcohol, and amines, different products can be recovered through glycolysis, hydrolysis, methanolysis and so on as shown in **Figure** **8**. With respect to the products via solvolysis, terephthalic acid (TPA), dimethyl terephthalate (DMT), and bis(hydroxyethyl)terephthalate (BHET) can be reused as precursors to re‐make PET, while other products such as, dioctyl terephthalate (DOTP) and 1,4‐benzene dicarboxamide can be used as plasticizer or chain extender for industrial chemistry.

#### 4.3.1 Glycolysis for Bis(hydroxyethyl)Terephthalate Recycling

**Figure 9:**
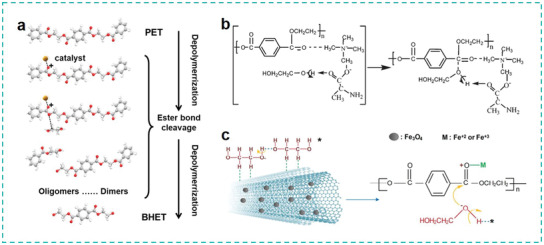
Gycolysis of PET with different catalyst for BHET recycling. a) Pathway of the conversational transition metal salt catalyzed depolymerization of PET. Color code: C, gray; O, red; H, White; metal, Yellow. b) Mechanism of [N1111][Ala] boosted glycolysis of PET. Reproduced with permission.^[^ ^138^ ^]^ Copyright 2012, Royal Society of Chemistry. c) Mechanism of the Fe_3_O_4_ MWCNT catalyzed glycolysis. Reproduced with permission.^[^ ^144^ ^]^ Copyright 2016, Royal Society of Chemistry.

Among these strategies, glycolysis is the most classical industrial process for recycling PET wastes, which is conducted at 180–250 °C with the presence of catalyst and excess glycol as solvent.^[^ ^134^ ^]^ Since using BHET rather than TPA or DMT as precursor for PET production can circumvent an intermediate process step, recovering high purity BHET from waste PET becomes the primary goal. Ethylene glycol (EG) is the most frequent used regent for PET depolymerization, where the depolymerization pathway is shown in **Figure** **9**a. Typically, the ester bonds of PET are activated by the interaction between Lewis acid‐like catalyst and the carbonylic oxygen resulting in that more electrons are dragged toward carbonylic oxygen atom. Subsequently, more partial positive charges are introduced toward the carbonylic carbon, which enhances the electrophilicity of carbon atom. The more electrophilic carbonylic carbon are more vulnerable to be attacked by the lone pair electrons of nucleophilic EG, which lead to the cleavage of ester bonds through the formation of new C—O bonds between carbonylic carbon and oxygen of the EG and break of the exiting C—O bonds of the PET. As the consequence, the continuous ester bonds cleavage give rise to the depolymerization of PET into BHET. However, the conversion of oligomers and dimers into BHET is reversible, meaning that when the monomers accumulation reaches a certain amount, the depolymerization reaction can shift backward. This phenomenon leads to the increasing amount of dimers and oligomers at the expense of the BHET.

In order to enhance the BHET selectivity, metal salts, ionic liquids, and heterogeneous catalysts have been applied as catalyst during the depolymerization of PET. Typically, the performances of metal acetate employed as homogenous catalyst in glycolysis have been extensively studied. However, the immiscibility of PET with the polyols leads to the limited glycolysis kinetics. In this context, Liu et al. developed a ultrafast glycolysis with maximum depolymerization efficiency of 100% and BHET yield of 83% were delivered at 190 °C within 1 min by using Zn(OAc)_2_·2H_2_O as catalyst and DMSO as cosolvent.^[^ ^135^ ^]^ The authors speculated that hydrogen bond between DMSO and PET played a key role in PET dissolution. However, the toxic DMSO, as well as, transition metal ions (Zn^2+^, Co^2+^, Mn^2+^) existing in the liquid residue can lead to the secondary pollution. To circumvent this problem, the non‐toxic sodium carbonate was studied by López‐Fonseca et al. as the green candidate for PET glycolysis, which exhibited a BHET yield of 80% at 196 °C within 1 h.^[^ ^136^ ^]^ Ionic liquids (ILs) are also a category of efficient catalyst for the conversion of PET into BHET, where the ILs can be easily separated from the final products. In 2009, Wang et al. first employed a neutral ionic liquid catalyst, 1‐butyl‐3‐methylimidazolium chloride ([bmim]Cl) for PET glycolysis. Interactions between cation and carbonyl oxygen, anion, and hydroxyl group of EG, respectively, could facilitate the nucleophilic attack on the carbonyl of PET and thus boosted glycolysis process. As the consequence, the maximum performances of 100% PET conversion and 70% BHET selectivity were delivered at 195 °C and 1 atm within 8 h.^[^ ^137^ ^]^ Later, Wang et al. developed acid tetramethylammonium alanine ([N1111][Ala]) achieving an outstanding glycolysis efficiency of 100% PET conversion and 74.3% BHET selectivity at 170 °C and 1 atm within 50min.^[^ ^138^ ^]^ The authors indicated the mechanism of the [N1111][Ala] catalyzed degradation, where the H‐bonds formed between EG and [Ala]^−^, [N1111]^+^, and PET, as well as, [N1111]^+^ and [Ala]^−^ led to the disconnection of long chain of PET and the acceleration of glycolysis (Figure 9b). In the last few years, various IL catalysts comprised of metal halide or metal acetate anions (i.e., Fe, Co, Cu, Zn) and [bmim] cation were studied,^[^ ^139^, ^140^ ^]^ which exhibited superior selectivity for BHET (>80% within 1 h) as well as, the recyclability than metal‐free ILs. More recently, choline formate ([Ch][For]) and choline acetate ([Ch][OAc]) with less toxicity were developed to circumvent the utilization of toxic imidazolium cations, which delivered competitive glycolysis activity (>80% in 3h).^[^ ^141^ ^]^ Further investigations will mainly focus on the development of novel catalysts possessing the merits of high activity, selectivity, environment friend, and recyclability.

Heterogeneous catalysts have been also studied to boost the transesterification reaction between PET and EG, which possess the advantage of the ease of separation. Shukla et al. utilized *β*‐zeolite and *γ*‐zeolite as first examples of heterogeneous catalysts for PET glycolysis, which exhibited similar efficiency of fully PET conversion and ≈65% BHET selectivity at 196 °C within 8 h.^[^ ^142^ ^]^ In order to enhance the separation from final products, magnetic catalysts have been developed. Kim et al. reported an efficient glycolysis of PET by first using EG as regent and superparamagnetic *γ*‐Fe_2_O_3_ nanoparticles as catalyst, affording a high yield (>90 wt%) of BHET within 1 h at 300 °C with the catalyst dosage of 5 wt%.^[^ ^143^ ^]^ The HPLC measurement shown that the BHET monomer account for the main glycolysis product. The superiority of *γ*‐Fe_2_O_3_ was further indicated by using ZnO, Mn_3_O_4_, and CeO_2_ as catalyst in the same glycolysis condition. Meanwhile, benefiting from the excellent thermal and chemical stability of the as‐synthesized *γ*‐Fe_2_O_3_ catalyst, the BHET transformation capacity of first 10 runs was not affected, which further proved the reusable of the catalyst. ElMetwally et al. designed a Fe_3_O_4_‐boosted MWCNT catalyst to enhance the BHET selectivity.^[^ ^144^ ^]^ As a result, a remarkably 100 wt% yield of BHET was achieved within 2 h at 190 °C by using the Fe_3_O_4_/MWCNT catalyst, where the catalyst could be conveniently recycled and its performance was steady after 8 glycolysis cycles. Importantly, the remarkably reduced processing temperature was beneficial for saving the energy input. The mechanism of the Fe_3_O_4_‐boosted MWCNT catalyst was illustrated in Figure 9c: Fe_3_O_4_ served as a Lewis acid‐like catalyst to trigger the scission of ester bonds, while the presence of MWCNT facilitated hydrogen bonding between with EG and an increase of the electronegativity of hydroxyl functional groups of EG. Thus, the depolymerization reaction was significantly promoted by the facilitated ester bonds cleavage.

Apart from EG, diethylene glycol, propylene glycol, dipropylene glycol, and other dihydric alcohols have been studied as solvents for PET glycolysis. However, the utilization of appropriate catalyst to promote the transesterification reaction between PET and EG for enhancing BHET selectivity is still the primary consideration for glycolysis, which could be ascribe to the BHET is the desirable precursor to re‐make PET in accordance with the concept of circular economy.

#### 4.3.2 Other Solvolysis for Fine Chemicals Recycling

Methanol is adopted to depolymerize PET for the recovery of DMT and EG, where the methanolysis is normally conducted at high temperatures (180–280 °C) and high pressures (20–40 bar). Similar to the glycolysis, the interaction between ester bonds and the lone pair electrons of nucleophilic methanol can result in the cleavage of ester bonds and depolymerization of PET. Therefore, the metal salts, ionic liquids and heterogeneous catalysts for boosting methanolysis have been reported,^[^ ^139^, ^145^, ^146^, ^147^ ^]^ where the design and choose of catalysts is similar to these of glycolysis. Sharma et al. also indicated the employment of cosolvents can promote the methanolysis process.^[^ ^148^ ^]^ Nevertheless, the presence of water impurities can perturb the transesterification process by poisoning catalysts, which gives rise to the shift of the methanolysis products to start with TPA instead of DMT.^[^ ^149^ ^]^ Although yielded DMT (>85% within 2 h) possessing high quality identical to virgin products gives the chance of integrating the methanolysis in the polymer production line, the toxicity of methanol, the cost‐consuming processes of separating, and refining the mixture products, as well as, conversing DMT into TPA and subsequent BHET for re‐making PET limit the industrial application of methanolysis.

Hydrolysis can be performed to recover TPA and EG using water as regent at high temperatures (180–300 °C) and high pressures (1–4 MPa) under acidic, alkaline, and neutral conditions. Although concentrated phosphoric, nitric, and sulfuric acids can be used as solvents to achieve high conversion, separating EG from the highly acidic solution remains a challenge. Alkaline hydrolysis is also a promising strategy to recycle TPA and EG from PET wastes with high efficiency, where the PET conversion of up to 100% could be delivered employing 4–20 wt% NaOH aqueous solutions as solvent.^[^ ^150^ ^]^ Compared to the acidic hydrolysis, longer reaction times (3–5 h) and higher temperatures (>200 °C) lead to the increasing energy input during the industrial production. Additionally, the extra utilization of acids and alkalis during hydrolysis can cause a series of economic and environmental problems. While water is the ideal solvent from the perspective of sustainability, the relatively weak nucleophilicity of water in the neutral hydrolysis reaction results in comparatively low hydrolysis efficiency.^[^ ^151^, ^152^, ^153^ ^]^

In addition to monomer recovery, PET also can be converted into fine chemicals via alcoholysis, aminolysis, ammonolysis, and so on, which can be used as raw materials for chemical industry. Zhang et al. reported an efficient alcoholysis route to convert PET into DOTP, which is a novel plasticizer exhibiting good cold‐ and thermo‐tolerance and chemical resistance with a wide scope of industry applications.^[^ ^154^ ^]^ In this study, 2‐ethyl‐1‐hexanol (2‐EH) and choline chloride‐based deep eutectic solvents (ChCl‐based DESs) were used as solvent and catalysts, respectively. Almost 100% PET conversion and 84.7% DOTP yield were obtained. Based on DFT calculations and experimental results, the authors believed that hydrogen bonds formed between 2‐EH and DESs plays a significant part to boost depolymerization as indicated by the lengthening of the O—H bond of the hydroxyl group in 2‐EH, which is more prone to be cleaved.

### 4.4 Cracking of Plastic Wastes for Energy Recovery

Cracking is a thermal decomposition process that occurs under an oxygen‐free atmosphere and is most widely used currently exploited to convert plastic wastes into energy. Generally, the products of pyrolysis have complex compositions with a distribution of molecular weights, attributed to the random chain scission during this thermal‐trigger decomposition process. A general mechanism is: The random scission of C—C bonds in the polymer chains at moderate to high temperature (e.g., ≈500 °C) which results in the formation of a number of radicals of varied chain lengths;^[^ ^155^, ^156^ ^]^ subsequently, these carbon radicals trigger a series of chain reactions including hydrogen transfer and *β*‐elimination;^[^ ^157^ ^]^ thus, the plastic wastes are thermally converted into low molecular weight organics such as the liquid fuels and gaseous substitute natural gas. In addition, the condensation of the intermediates (e.g., olefins, cycloalkanes, aromatic hydrocarbons, etc.) will result in the formation of macromolecular PAHs, tar, and coke. Previous studies showed that even for the cracking of polyolefin‐type plastics with the simplest molecular structure (i.e., the PE or PP) at relative low processing temperature (<450 °C), the random fragmentation results in the difficulty to obtain narrow distributed products.^[^ ^158^, ^159^ ^]^ As a consequence, a series of hydrocarbons possessing carbon numbers ranging from C_1_ to C_20_, including alkanes, alkenes, polyene hydrocarbon, alkynes, cycloalkanes, aromatic hydrocarbons, and their isomers could be detected in the cracking products. This phenomenon gives rise to the increase recovery costs for the additional separation and purification operations to yield naphtha (alkanes in C_5_–C_7_ range), gasoline (aliphatic hydrocarbons and aromatic hydrocarbons in C_4_–C_12_ range) or diesel (alkanes, naphthenes, and aromatic hydrocarbons in C_10_–C_22_ range) and other high quality liquid products. In this context, aiming to decrease the complexity of products, various strategies have been developed to control the random fragmentation, in which the catalytic cracking, catalyst‐free pyrolysis (thermal cracking), hydrogenolysis, and advanced technologies assisted pyrolysis are the current research hotspots.

#### 4.4.1 Catalytic Cracking

Catalysts are critical ingredients for modern chemical engineering process, which are capable to reduce the reaction temperature and/or absorb and release certain intermediates during cracking, achieving the energy conservation and the control of product distribution for enhancing the quality of final products. Natural zeolites, artificial molecular sieve, and other solid acid‐based catalysts possessing Lewis acid or Brønsted acid feature have been applied as catalysts for this technique. Typically, the mechanism for these Lewis acid catalysts is as follows: The active sites of these Lewis acids serve as electrons acceptors for carbon atoms of plastic to form carbocations in plastic chains, which promote the formation of isohydrocarbons and aromatic hydrocarbons. Similarly, researchers pointed out that the Brønsted acids catalyzed cracking of plastics is also triggered by carbocations, whereas they believe the formation of carbocations is ascribed to protons donation from Brønsted acids to carbon atoms of plastics.

Previous works focused on utilizing zeolites, such as, ZSM‐5 and USY, as traditional thermal catalysts for the cracking of PE plastics, whereas the low product selectivity gave rise to the formation of various liquid and gaseous products with wide carbon number range.^[^ ^160^, ^161^ ^]^ The as‐obtained liquid with complex components cannot be directly utilized as naphtha, gasoline, or diesel. Therefore, the wide distribution of products needs to be separated and purified via additional processes to yield high quality fuels, where these energy consumption and time‐consuming operations are main challenge for further development of catalytic cracking. Serrano et al. studied the catalysis efficiency of an aluminum‐substituted mesoporous silicon catalyst Al‐MCM‐41 to crack LDPE for yielding hydrocarbons.^[^ ^145^ ^]^ However, the shape selectivity of Al‐MCM‐41 with enlarged pores size was not enhanced, where a wide range of hydrocarbons up to C_20_ were obtained after 2 h for catalytic cracking at 420 °C. Meanwhile, Al‐MCM‐41 is an expensive catalyst, since special organic structural‐directing agents were used in the fabrication process. Therefore, developing effective and low‐cost thermal catalysts to convert LDPE into more valuable gasoline‐type hydrocarbons via a facile route is still a huge challenge.

Rational design and fine tuning the properties of the catalyst can potentially enhance the selectivity and productivity of this process. Acidity adjustment and enhancement of their catalytic stability by enlarging the surface area for more efficient mass diffusion are currently the focus for researchers. Recently, Zhang et al. reported a one‐step strategy to selective yield gasoline‐type product with a distribution between C_4_ and up to C_10_ at 250–350 °C by using a well‐defined aluminum‐incorporated SBA‐15 (Al‐SBA‐15) catalyst.^[^ ^162^ ^]^ This catalyst displayed a superior shape selectivity endowed by its proper pore size (8.8 nm), which provided sufficient voids for the penetration of the LDPE chains thus suppressing the occurrence of secondary re‐cracking reactions during the cracking. Thus, sufficient and homogeneous Brønsted sites introduced by aluminum with modulated strength ensured a moderate cracking, offering a high selectivity toward C_4_–C_10_ products for liquid fuels recovery. Moreover, the porous structure with appropriate pore size also facilitates the diffusion and release of gasoline‐like hydrocarbons, which effectively suppresses the generation of gas products through the undesirable secondary re‐cracking reactions due to accumulation and re‐adsorption of these liquid products.

#### 4.4.2 Catalyst‐Free Pyrolysis Under Hydrothermal Process

Generally, the pyrolysis of plastic wastes under 450–800 °C with the absence of catalysts can yield low‐quality liquid products with wide molecular weight distribution, where the high molecular weight waxes (in C_18_–C_30_ range) are the major products. Nevertheless, it is a cost‐consuming process to convert these as‐produced downgraded oil products into fuels. Hydrothermal liquefaction (HTL) is an efficient catalyst‐free strategy for the conversion of plastic wastes to high quality liquid products developed in recent years. Supercritical water obtained in a sealed reactor at over 374 °C and 22.1 MPa can be used as solvent and reactant (or catalyst) during this process. A typical procedure is, 1) the molten polymer partially dissolved into the supercritical water results in the dilution of the polymer phase, in which the polymer decomposition is promoted and the pyrolysis selectivity is shifted from bimolecular hydrogen abstraction and addition to unimolecular *β*‐scission; 2) the proton‐transfer reactions between supercritical water and polymer through hydrogen bonding endow it acid feature boosting the formation of carbocations, which serve as initiators to trigger the C—C cleavages in polymer chains.^[^ ^163^, ^164^ ^]^ Thus, the development of supercritical water‐assisted pyrolysis can not only shift the products selectivity from high molecular weight waxes into lower molecular weight fuels, but also open cost‐effective way by circumventing the use of catalyst.

**Figure 10:**
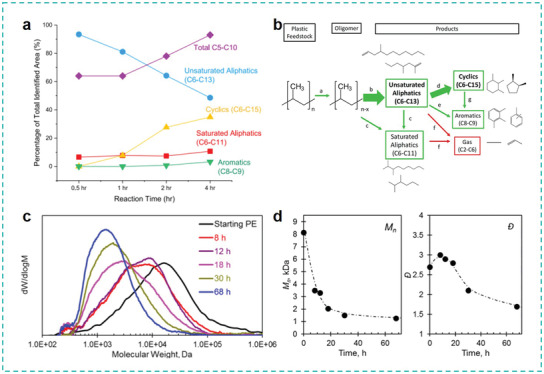
Cracking of plastic waste for energy recovery. a) Quantification analysis of products within 4 h at 425 °C. b) Proposed mechanism of converting PP into liquid fuels via the supercritical water assisted HIL process. Reproduced with permission.^[^ ^165^ ^]^ Copyright 2019, American Chemical Society. c) Weight distribution, d) *M*_n_ and Đ plots of the hydrogenolyzed products over Pt/SrTiO_3_ catalyst. Reproduced with permission.^[^ ^170^ ^]^ Copyright 2019, American Chemical Society.

Wang et al. reported a HTL process to recycle PP into valuable fuels assisted by supercritical water at 380–500 °C and a high pressure of 23 MPa.^[^ ^165^ ^]^ The oil products (in C_4_–C_15_ range) were obtained with a fairly high yield of over 90 wt% at 425 °C, 2–4 h and 450 °C, 0.5–1 h, possessing similar heating values (46.3–49.3 MJ kg^−1^) as gasoline (47.7 MJ kg^−1^). In particular, the maximum gasoline (C_5_–C_10_) selectivity of over 80 wt% versus the total products was delivered at 425 °C, 4 h (**Figure** **10**a). Interestingly, the formation of coke was suppressed to <1 wt% via the supercritical water‐assisted pyrolysis at moderate temperature, which is much lower than that of (up to 18 wt%) in traditional pyrolysis.^[^ ^166^ ^]^ The authors also stated that the reaction temperatures >450 °C or the reaction times longer than 4 h should be circumvented to suppress the generation of undesirable gaseous products. Based on the analysis of the major intermediates obtained at various experiment conditions, the reaction pathways were proposed by the authors (Figure 10b): When the reaction temperature reaches 425 °C, PP was quickly depolymerized into oligomers within 0.5 h; extending further the reaction time afforded a product comprised with different cyclic compounds, which were expected to form through the cyclization of unsaturated aliphatics; simultaneously, a small percentage of unsaturated aliphatics were converted into saturated aliphatics and aromatics. Dehydrogenation of cyclics was assumed to afford the aromatics. It was also found that the side chains (CH—CH_3_ bonds) of PP are more prone to cleave due to their relatively lower bonding energy,^[^ ^164^ ^]^ affording a large amount of radicals, which are the critical precursors to yield olefins through *β*‐scission. Apart from the study of supercritical water‐assisted PP pyrolysis, PE plastics have been successfully converted into oil product with high heating values, in which the maximum productivities of 98 wt% is delivered.^[^ ^167^ ^]^

#### 4.4.3 Hydrogenolysis

Hydrogenolysis is described as a promising protocol to break down high molecular weight polyolefins into valuable liquid products (i.e., gasoline and lubricant), which undergo the C—O and/or C—C bonds cleavage and simultaneous addition of hydrogen. This process usually proceeds at 150–400 °C in a H_2_ atmosphere with a H_2_ pressure of 1–10 MPa in the presence of catalysts.^[^ ^168^ ^]^ Normally, the catalyst for hydrogenolysis is comprised of two pieces: 1) Transition metals (Pt, Ni, Mo, Fe) sites for hydrogenation; 2) Lewis acid (zeolites, artificial molecular sieves, and perovskites) sites for C—C bonds cleavage. Taking PE as the example, appropriately designed catalyst is a prerequisite to selectively absorb higher molecular weight PE chains and facilitate the internal C—C bonds cleavage to yield liquid hydrocarbon with specific chain lengths. Nevertheless, previous work figured out the excessive hydrogenolysis resulting from the strongly interactions between polymer and catalyst would lead to the formation of light hydrocarbons such as, methane and ethane.^[^ ^169^ ^]^ Therefore, the hydrogenolysis of polymers is inclined to yield wide distribution products.

Aiming at tuning the products distribution, Delferro et al. demonstrated a selective hydrogenolysis process by transforming PE into high‐quality liquid hydrocarbon products (i.e., motor oil and waxes), where the SrTiO_3_ perovskite nanocuboids decorated with well‐dispersed Pt nanoparticles (2.0 ± 0.5 nm, 11.1 wt% Pt loading) were used as the catalysts.^[^ ^170^ ^]^ Since the surface property of SrTiO_3_ was adjusted by the decoration of Pt nanoparticles, the interactions between SrTiO_3_ and polymers were weakened. Therefore, the formation of small molecules through excessive hydrogenolysis was suppressed. Under a solvent‐free catalytic hydrogenolysis (1.17 MPa H_2_ and 300 °C), the Pt‐SrTiO_3_ catalyst completely converted pristine PE into low‐molecular products with decreased *M*_n_, *Đ* and narrow molecular weight distribution (Figure 10c,d). After hydrogenolysis for 96 h, lubricant‐like products of narrow *M*_w_ distribution (*M*_n_ = 590 Da, *M*_w_ = 625 Da, and *Đ* = 1.1) were obtained with a yield of 42 wt% from a PE raw material (*M*_n_ = 8150 Da, *M*_w_ = 22 150 Da, and *Đ* = 2.7). In addition, high‐quality liquid products (*M*_n_ = 990 Da, *M*_w_ = 1130 Da, and *Đ* = 1.3) with yield of 97% was also produced by using the commercial‐grade disposable plastic bags as the starting materials (*M*_n_ = 33 000 Da, *M*_w_ = 115150 Da, and *Đ* = 3.5) under the same reaction condition. Interestingly, the weight distribution with the increasing reaction times illustrated the inclination to yield narrow molecular weight products, suggesting high molecular weight chains were more prone to undergo hydrogenolysis than that with low molecular weight. Furthermore, DFT calculation demonstrated that the total adsorption energy of *n‐*alkanes on Pt sites increased with the number of carbon atoms, which was consistent with excellent product selectivity. Moreover, the authors indicated the interaction between Pt and SrTiO_3_ substrates could stabilize the catalytic sites by avoiding the growth or aggregation of Pt nanoparticles at high temperature, thus ensuring the catalytic performance during hydrogenolysis over a long reaction time. Moreover, the size effect of the Pt nanoparticles could influence the product selectivity, where undesired light hydrocarbons were formed through over hydrogenolysis triggered by smaller Pt nanoparticles. More recently, taking account into the growing demand and the high value of lubricants, Kots et al. proposed an interesting upcycling strategy to obtain high‐quality lubricants from PP wastes.^[^ ^171^ ^]^ A cheaper and commercially available catalyst, ruthenium deposited on titania (Ru/TiO_2_), was used to selectively convert PP plastics into valuable lubricant‐range hydrocarbons with narrow molecular weight distribution at low temperatures of 250 °C with a modest H_2_ pressure of 3 MPa. A high lubricants yield of 66–80% within 16 h were delivered by using different PP products including amorphous polypropylene, everyday bags, and bottles as precursors.

### 4.5 Pyrolysis of Plastic Wastes for Generating Functional Carbon Materials

In recent years, the development of functional carbon materials has been focused on addressing contaminant absorption, energy storage, energy conversion, and environmental remediation.^[^ ^172^, ^173^, ^174^, ^175^, ^176^, ^177^, ^178^ ^]^ Converting plastic wastes into high added‐value functional carbonaceous materials through pyrolysis is a promising strategy whereby the C—C bonds based polymer structure can be reformed into solid carbonaceous materials. Typically, this process is operated at high temperature (>500 °C) under oxygen‐free atmosphere (vacuum, or utilizing inert N_2_ and Ar). Large amounts of volatile short‐chain aliphatic hydrocarbons are generated through the decomposition of plastic wastes at the primary stage of pyrolysis; these volatile organics were subsequent cyclized into cycloparaffins, which are further converted into alkylbenzenes and multi‐ring aromatics through dehydrogenation and polycondensation; as a result, the solid carbonaceous material with amorphous or partial graphitized structure is obtained as the final product. Through rational structural design of the morphology (specific surface area and porosity), chemical composition (heteroatom doping elements and configurations) of the carbon materials can be also effectively regulated by adjusting reaction conditions (i.e., maximum temperature, holding time, atmosphere, catalysts). Thus far, various functional carbon materials, such as, 2D graphene‐based materials, carbon spheres and porous carbon flakes (PCFs), have been successfully synthesized by using PE, PP, PS, and other plastic wastes as precursors.^[^ ^179^, ^180^, ^181^, ^182^, ^183^ ^]^

Typically, traditional pyrolysis for transforming plastic wastes into functional carbons is comprised of two stages. First, the plastic wastes are heated at a relative low temperature (400–600 °C) for primary pyrolysis to obtain the amorphous or partial graphitized carbons, which is the so‐called pre‐carbonization. Subsequently, the further pyrolysis is processed at higher temperature with the presence of chemical agents such as pore‐forming agents, template agents for adjustment of morphology to endow the carbon materials certain functions, which is known as the activation. In addition to the inert gases, the reactive H_2_, CO_2_, and NH_3_ atmosphere can be utilized for introducing the defects.

**Figure 11:**
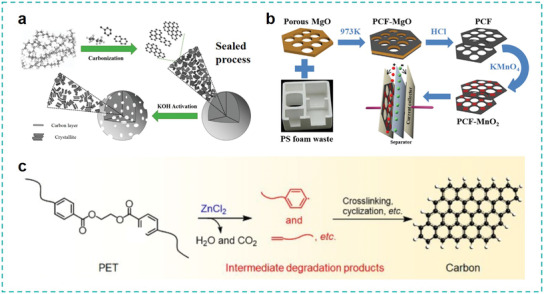
Synthesis of functional carbon materials derived from plastic wastes. a) Schematic illustration for the formation of HPCs via autogenic pressure carbonization and KOH activation. Reproduced with permission.^[^ ^180^ ^]^ Copyright 2019, American Chemical Society. b) Scheme of the fabrication of PCF and PCF‐MnO_2_ hybrid. Reproduced with permission.^[^ ^181^ ^]^ Copyright 2019, Elsevier. c) ZnCl_2_/NaCl eutectic salt assisted pyrolysis strategy for HPCs synthesis. Reproduced with permission.^[^ ^183^ ^]^ Copyright 2019, Royal Society of Chemistry.

Potassium hydroxide (KOH) is a classical activation for synthesizing porous carbons, which can etch carbon at high temperature to generate porous structures. Owing to the strong reaction between carbon and KOH, it is advised that the precursor should be pre‐carbonized before the co‐pyrolysis with KOH rather than a one‐pot co‐pyrolysis to improve the productivity of functional carbons. He et al. reported a two‐stage pyrolysis strategy to synthesize a carbon material with hierarchical porous structure from LDPE (**Figure** **11**a).^[^ ^180^ ^]^ The first step was processed in a sealed reactor at 600 °C without using any catalyst, which could enhance the graphitization process by facilitating the aromatization and polycondensation of the as‐generated small molecules. At the first stage, 45 wt% carbonaceous products with micrometer‐scale spherical morphology were obtained. The next‐stage activation process was assisted by KOH at 700 °C, affording the carbon product (HPC) with hierarchical pores, large specific surface area (3059 m^2^ g^−1^) and abundant surface functional groups. For the application in symmetric supercapacitor, the hierarchical porous carbon electrode exhibited a maximum specific capacitance of 355 F g^−1^ at a current density of 0.2 A g^−1^, a maximum energy density of 9.81 W h kg^−1^ and power density of 450 W kg^−1^ at 1 A g^−1^ in 6 m KOH electrolyte. The authors explained that the superior electrochemical performance could be ascribed to the boosted double‐layer capacitance and pseudocapacitance through enhancing surface adsorption of electrolyte ions and facilitating redox reactions of surface functional groups.

Template agents can be used through a one‐pot pyrolysis to simplicity and efficiently construct hierarchically carbonaceous framework. Tang et al. demonstrated a protocol to fabricate functional PCF by direct pyrolysis of PS wastes using porous MgO as the hard template at 700 °C (Figure 11b).^[^ ^181^ ^]^ The as‐prepared carbon flakes are characterized as hierarchical porous morphology, high specific surface area (1087 m^2^ g^−1^) and high conductivity, which can be used as a conductive substrate for energy storage. After the surface modification by MnO_2_ possessing native high specific capacity, the PCF‐MnO_2_ electrode delivered superior electrochemical performances of ultrahigh capacitance of 308 F g^−1^ at 1 mV s^−1^ and 247 F g^−1^ at 1 A g^−1^ in LiCl electrolyte, and steady capacitance retention of 93.4% after 10 000 cycles at 10 A g^−1^ in symmetric supercapacitors. Li et al. synthesized 2D‐thin carbon film coated SiO_2_‐based glass fiber membrane by using linear PE as the carbon source.^[^ ^182^ ^]^ The thin PE derived carbon film was successfully coated on GF membrane at 900 °C, where the PE and GF were put in two different silica boats side by side in a tube muffle furnace. The modified membrane with 2D‐thin carbon film remarkably enhanced the optical absorption and solar thermal conversion capability comparing with the pure glass fiber membrane. After the further modification by polydopamine (PDA) to increase the hydrophily, the functional membrane exhibited superior evaporation rate and energy transfer efficiency of 1.39 kg m^−2^ h^−1^ and 80.4% under simulated sunlight irradiation with 1 sun.

Polyesters with heteroatomic (such as, oxygen and nitrogen) backbone can also be used as the substrates for developing heteroatom doped carbon materials. Compared with the almost fully carbon‐based polyolefins, polyesters are featured by the presence of additional oxygen atoms in their ester groups. These oxygen species are potentially a source of heteroatoms and can be doped in the carbon framework during pyrolysis. Gong et al. reported a ZnCl_2_/NaCl eutectic salt‐assisted pyrolysis strategy to synthesize carbon material with inter‐connected hierarchical porous morphology from PET wastes (Figure 11c).^[^ ^183^ ^]^ The dehydration and decarboxylation of PET could be boosted by ZnCl_2_, where the as‐formed vinyl‐terminated chain fragments and aromatic rings could further undergo cyclization and crosslinking to construct carbon framework. Meanwhile, ZnCl_2_/NaCl eutectic salt acts as the pore‐forming agent at 550 °C, affording a nanoparticle product with inter‐connected hierarchical structure. The as‐prepared hierarchical porous carbon material (HPC) exhibited high specific surface area (776.2 m^2^ g^−1^) and large pore volume (0.663 cm^3^ g^−1^), endowing them high solar steam generation performance via the enlarged connect interface between functional carbon and water. Moreover, these particles also have abundant oxygen‐containing functional groups (—OH and —COOH), which is beneficial to facilitate the diffusion and transportation of water in the systems and thus boost the efficiency of solar steam generation. Because of a combination of such a porous structure and oxygen doping, the obtained functional carbon materials delivered a high water evaporation rate of 1.68 kg m^−2^ h^−1^, a high solar‐to‐vapor conversion efficiency of 97%, ca. 99.9% metallic ion removal efficiency and >99.9% dye removal efficiency of sea water, thus are promising to be applied for solar steam generation or water treatment application.

### 4.6 Novel Strategies for Process Engineering Control

**Figure 12:**
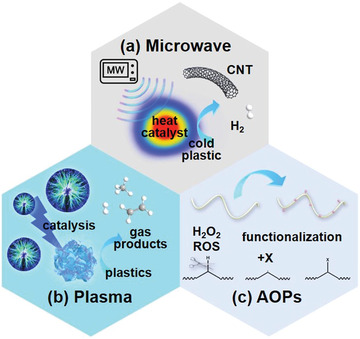
Novel strategies for process engineering control: a) Microwave; b) Plasma; c) C—H activation by AOPs.

Taking into account the drawbacks of the conventional solvolysis and pyrolysis, the relatively low thermal conductivity of plastics leads to severe thermal balance in industrial reactors. Meanwhile, the self‐decomposition of plastics (especially during pyrolysis) gives rise to the uncontrollable reaction pathways, yielding unselective products distribution by the unexpected side reactions. Recently, the techniques discussed above have been coupled with additional engineering protocols such as, microwave irradiation, plasma treatment, and ROSs to enhance reaction selectivity and to optimize the products distribution (**Figure** **12**).

#### 4.6.1 Microwave‐Initiated Pyrolysis

Microwave electromagnetic energy can be directly absorbed by matter with microwave‐absorbing feature, and subsequently converted into heat. Therefore, microwave has been used in domestic and industry applications given its fast and highly efficient heating capability. The absorption of microwave energy is notably strong for sp^[^ ^2^ ^]^ hybridized carbons or metals, making it a powerful tool for degradation of certain plastics. As reported by Voiry et al, very high temperature up to even several thousand of Celsius could be obtained by utilizing graphene as microwave‐absorbing catalyst under microwave irradiation with ultrafast heating rate within only tens of milliseconds.^[^ ^184^ ^]^ When irradiating the mixture of catalyst and plastic wastes by microwave, a high temperature can be rapidly and selectively generated at the catalyst rather than heating the whole reactor, resulting in the cracking reaction mostly occurring at the interface between catalyst and plastic waste (Figure 12a). The generated gaseous products easily leave the reaction area, preventing the excessive cyclization, dehydrogenation, and other complex reactions. Therefore, microwave‐initiated pyrolysis has been considered as a promising protocol for the fast cracking of plastic wastes into gaseous short chain hydrocarbons.

Typically, liquid aliphatics and aromatics are the major products via conventional pyrolysis of polyolefins, while the gaseous products only account to 5–20 wt% of the plastic wastes input. Interestingly, the gaseous products selectivity can be enhanced by the microwave‐initiated pyrolysis. Qiao et al. developed a microwave‐excited “drop‐in” cracking protocol to selectively convert the mixed linear LDPE powder and palm oil (PO) into ethylene, propylene and other gaseous fine chemicals by continuously dropping the mixture onto the microwave acceptor in cracking system.^[^ ^185^ ^]^ Carbon‐coated aluminum oxide fibers (CAF) were used as microwave acceptor, which can quickly convert the microwave into heat leading to a fast increase of temperature to >1000 °C within 7 min under 900 W microwave irradiation. Due to high gaseous productivity (>70%) during the cracking of pure PO, the PO could serve as the appropriate solvent to transport the PE powder onto the surface of CAF with controllable rate and negligible impact on the gas selectivity during the cracking of PE‐PO mixture. A summed yield of 45 wt% of ethylene and propylene was obtained via cracking of PE‐PO mixture, comparable to that of naphtha produced through traditional steam cracking. The authors also claimed that other type of plastic wastes including PP, PET, and PS could be cracked into value‐added products by the same plant oil‐assisted “drop in” approach.

When the microwave acceptor contains catalytic components (i.e., Fe, Co, Ni‐based materials), the gaseous products generation at the catalyst/plastic interface can be in situ converted into carbon nanotubes. Edwards et al. proposed a novel microwave‐activated strategy to yield carbon nanotubes and H_2_ from HDPE plastic waste.^[^ ^186^ ^]^ With the presence of the mixture of iron oxide and aluminum oxide (FeAlO*_x_*) as the microwave acceptor, the HDPE plastic waste was efficiently converted into H_2_ with a high yield of 97% (55.6 mmol g_plastic_^−1^) within 20 s under 1000 W microwave. Remarkably, the only residue of this process was identified as high‐quality multi‐walled carbon nanotubes. The authors further proved that the fast cracking reaction mainly took place at the interface between microwave acceptor and plastics, rather than a homogeneous cracking of plastics in the traditional thermal cracking process. In this context, the side reactions caused by the decomposition of plastics were effectively suppressed hence significantly enhancing the selectivity.

#### 4.6.2 Plasma‐Cracking Coupling Technology

Cold plasma, which can generate highly energetic electrons, excited molecules and ions, is a novel room temperature synthetic technique featured by ultrahigh average electron temperature (10^4^–10^5^ K, 1–10 eV).^[^ ^187^ ^]^ Interestingly, reactive species (i.e., atomic oxygen, hydroxyl, and excited oxygen) generated by the collisions between high temperature electrons and the relatively cold gas molecules can trigger some thermodynamically unfavorable reactions, which are normally rather difficult to occur under traditional synthetic condition, by influencing their kinetics. Thus, the degradation of heavier hydrocarbon molecules into lighter hydrocarbons or fuels can be achieved by the more thorough cleavage of C—C and C—H bonds assisted by the powerful energetic electrons. In some cases, both the reactivity and product selectivity are dramatically improved for gaseous organics recycling (Figure 12b).

The cold plasma has been utilized to assist the pyrolysis for recovering ethylene (C_2_H_4_) from HDPE waste by Phan et al.,^[^ ^188^ ^]^ where the N_2_ plasma with specific energy density (the discharge power supplied to the cold plasma reactor per unit of gas volume) of 180 J mL^−1^ was adopted. The detailed plasma reactor illustrated the 316L stainless steel mesh exteriorly covered the outer tube and the stainless steel sheet interiorly placed in the inner tube served as the two electrodes to create a cold plasma zone. With the assistance of plasma, a yield of 24 wt% of C_2_H_4_ was achieved at 700 °C at a heating rate of 75 °C min^−1^, which is ≈55 times higher than that obtained without cold plasma treatment. The authors also found that the presence of a small amount of solid acid catalyst (HZSM‐5 and SO_4_^2−^—ZrO_2_) can further enhance the gas yield, which was able to promote the *β*‐scission, a kinetically dominating reaction step during the degradation cascade toward the formation of the desired ethylene product. In particular, a more oxidative atmosphere was created by catalyst through the transformation of plasma discharge type from a filament discharge into a hybrid discharge, meaning that both micro‐discharges and surface discharge inside the catalyst pores were both present in this process, thus favoring the formation of lighter compounds.^[^ ^189^ ^]^ Besides, the physical/chemical properties (i.e., surface area and electronic state) of catalyst were tuned by plasma, which could boost the catalytic activity and product selectivity during the cold plasma‐pyrolysis recovery process. With the synergistic effect of cold plasma and catalyst, the cold plasma‐catalytic pyrolysis coupling technique has the potential to be utilized for the large scale application of converting plastic wastes into valuable chemicals for the advantages of simple process as well as high gaseous product selectivity.

White waste pollution has been exacerbated, as the surge in medical plastic wastes (such as, face masks and medical gloves) from Covid‐19. In this context, Xu et al. developed a carbon foam microwave plasma process to convert unsorted white wastes into gases (H_2_, CO, C_2_H_4_,C_3_H_6_,CH_4_, etc.) and solid carbon in a N_2_ atmosphere, where the plasma discharge were used to generate heat and thus trigger the decomposition of plastics.^[^ ^190^ ^]^ When exposing carbon foam to nitrogen gas and microwaves, a large electric field can be formed on the surface of the carbon foam through the excitation of itinerant electrons in the carbon foam by incident microwaves. This phenomenon can give rise to the discharge of gas plasma and ionization of nearby N_2_ gas, which heats the local zone to over 3000 K within a few seconds. As the consequence, the white wastes are decomposed by plasmas into hydrogen‐bearing small gas molecules. The excess high‐quality graphitic carbons are precipitating out and grafted onto the pre‐existing foam. Interestingly, electrically conductive barbed tips are formed through the grafting of solid graphitic carbon on the surface of the carbon foam, which facilitate further plasma discharge and thus deliver enhanced decomposition efficiency over time.

#### 4.6.3 Reactive Oxygen Species Treatment Assisted Chemical Recycling

Plastics are known to have excellent anti‐chemical properties. Their high hydrophobicity and the absence of surface functional groups result in an increased resistance to attacks by chemical or microorganisms.^[^ ^24^, ^191^ ^]^ AOPs are classical methods utilized under ambient temperature and pressure, in which various strategies have been developed to convert hydrogen peroxide, PMS, peroxydisulfate, and other peroxides into ROSs by catalysts, heat, microwave, ultrasound, UV, and electricity.^[^ ^97^, ^98^, ^192^, ^193^ ^]^ The as‐generated highly reactive ROSs, such as, ^•^OH (*E*_0_ = 2.7 V vs NHE) and SO_4_^•−^ (*E*_0_ = 3.1 V vs NHE) possessing high oxidation potential can non‐selectively break the chemical bonds of organics, exhibiting the capability to degrade a number of organic pollutants into small organics, even to CO_2_ and H_2_O.^[^ ^194^, ^195^ ^]^ Given that the synthetic plastics are also organic matters, AOPs have thus attracted attention recently, with the aim to convert the plastic wastes into value‐added chemicals under mild condition (Figure 12c).

Taking plastics as substrates, a hydrogen atom abstraction pathway induced by the interaction between plastics and ^•^OH leads to the scission of C—H bonds and thereby generation of carbon‐centered radicals on the polymer backbone.^[^ ^196^ ^]^ Subsequently, *β*‐scission and chain transfer of these carbon‐centered radicals give rise to the random C—C backbone cleavage and molecular weight reduction. In this situation, a series of small organic molecules are generated as intermediates, which can be further mineralized into CO_2_ and H_2_O. Therefore, it is challengeable to selectively convert the plastic wastes into desirable products through Fenton reaction‐triggered plastics degradation. Interestingly, Chow et al. demonstrated an innovative and mild approach to transform the used PE plastics into value‐added carboxylic acids under ambient conditions by combining chemical activation and Fenton reaction.^[^ ^197^ ^]^ After a pretreatment by chlorosulfuric acid, sulfonate groups (—SO_3_^−^) were introduced onto the inert PE, which were subsequently coordinated with the Fe (III) catalysts forming reactive Fe (III)–sulfonate (PESO_3_‐Fe) complex. The authors proposed that C—C bonds of PE adjacent to PESO_3_‐Fe became vulnerable to cleave during the following Fe (III) activied Fenton process and ultimately afford a mixture of carbon‐based degradation products. The qualitative and quantitative analysis of these products indicated that the dissolved organics take up about 73.3 wt% with the majority (87.5 wt%) of monocarboxylic, dicarboxylic acids and organic carboxylic acids with carbon number of 1–4, whereas the other 26.7 wt% was mainly CO_2_. Even though the Fenton reaction is known for its non‐selectivity, experimental results obtained in this work indicated the chlorosulfuric acid pre‐treatment assisted Fenton process could deliver selective transformation of PE wastes into valuable carbon resource.

During the thermal‐assisted recycling (including extrusion and pyrolysis) of PVC, hydrogen chloride, and other toxic products will be released, giving rise to the secondary pollution. In particular, HCl generated at low temperature (below 300 °C) can lead to serious corrosion of the reactor, while toxic chlorinated organics can be released at high temperature (above 300 °C).^[^ ^198^ ^]^ Since the separation of PVC from the mixed plastic wastes (such as, PET and PMMA) possessing similar hydrophobic surface and appearance densities is a challenge, the release of HCl during the mechanical recycling of PET steams with trace amounts of PVC impurity results in the degradation of PET and damage of equipment. To address this issue, Truc et al. further demonstrated a process where ROSs were utilized to selectively act on PVC waste to achieve a highly efficient separation from a mixture of plastic waste. The waste mixture was treated with H_2_O_2_ under ultrasonic condition (3% H_2_O_2_, ultrasonic at 30 °C for 30 min),^[^ ^199^ ^]^ where large amount of ^•^OH radicals and per‐hydroxyl radicals (HOO^•^) radicals were generated by the assistance of ultrasonic. Afterward, these ^•^OH radicals substituted the chlorine groups in PVC and converted it into a more hydrophilic oxidized product with a large amount of carboxyl (COOH) and carbonyl (C = O) groups. As the consequence, the dechlorinated PVC were facilely collected from the bottom of reactor, which could be ascribed to an enhanced density upon water absorption, while other plastics with negligible chance of wettability still floated in the water. This process successfully achieved a remarkable PVC separation efficiency of 100%.

### 4.7 Photoreforming

**Figure 13:**
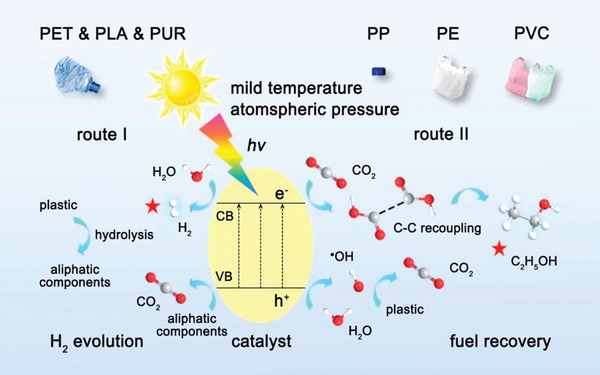
Routes to photo‐reform plastic waste: I) H_2_ evolution; II) fuel recovery.

Recently, photocatalytic technology utilizing renewable solar energy as power sources has been extensively explored in the fields of energy conversion and environmental remediation including CO_2_ reduction, water splitting, sea water desalinization, and organic pollutants removal.^[^ ^200^, ^201^, ^202^, ^203^, ^204^ ^]^ As mentioned in Section 3.6, the photo‐excited holes (h^+^_VB_) generated on catalyst under light with suitable wavelengths can transform H_2_O into ^•^OH, which serves as the significant species degrading organic pollutants into short‐chain organics and finally CO_2_ and H_2_O. Recently, the degradation of plastics into CO_2_ and H_2_O utilizing ZnO, TiO_2_, and other semiconductors as photocatalysts has been widely studied, in which the highest mineralization efficiency of nearly 100% has been reported.^[^ ^112^, ^113^, ^205^, ^206^ ^]^ Aside from traditional photocatalytic mineralization of plastic wastes into H_2_O and CO_2_, the conversion of plastic wastes into value added chemicals by photoreforming is a newly emerging field with huge economical potential (**Figure** **13**). Nevertheless, the photoreforming technology aiming to utilize plastic wastes as carbon sources for valuable chemicals recovery were seldom explored.

**Figure 14:**
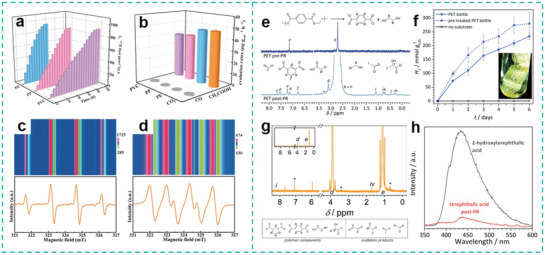
Photocatalytic conversion of plastics into fuels and small molecular. a) The CO_2_ yield of pure PE, PP, and PVC over the Nb_2_O_5_ catalyst during the photocatalytic conversion. b) Evolution rates of CH_3_COOH during the photocatalytic conversion of pure PE, PP, and PVC. c,d) In situ ESR spectra of DMPO−OH adducts formed during the photocatalytic conversion of PE using pure water and methanol as the solvent. Reproduced with permission.^[^ ^207^ ^]^ Copyright 2020, Wiley‐VCH. e) Mass spectra of the gas evolved after photoreforming. f) Long‐term photoreforming of a PET bottle to H_2_ using CdS/CdO*_x_* QDs under simulated sunlight. Reproduced with permission.^[^ ^208^ ^]^ Copyright 2018, Royal Society of Chemistry. g) ^1^H NMR spectra of after photoreforming and corresponding chemical structures and peak assignments. h) Emission spectra of pure 2‐hydroxyterephthalic and terephthalic acid after photoreforming. Reproduced with permission.^[^ ^209^ ^]^ Copyright 2019, American Chemical Society.

A complete mineralization and following photoreforming strategy was applied to convert plastic waste into high‐energy‐density C_2_ fuels. Xie et al. reported a photo‐induced reforming process under a simulated natural environment with visible light, where PE was first completely converted to CO_2_ and H_2_O, and C—C bonds coupling occurred in a sequential reactor to convert CO_2_ into CH_3_COOH.^[^ ^207^ ^]^ When using layered Nb_2_O_5_ catalyst with single‐unit‐cell thickness (≈3 nm) as the photocatalyst, a complete mineralization of PE was achieved within 40 h with a CO_2_ evolution rate of 23.1 mg g_cat_^−1^ h^−1^(**Figure** **14**a). Based on the qualitative experiments (Figure 14c,d), a large amount of ^•^OH radicals were produced by oxidation of H_2_O at the photoexcited holes in the valence band of Nb_2_O_5_, triggering the cleavage of C—C bond and resulted in a complete mineralization of PE. Meanwhile, the reduction of O_2_ by photoexcited electrons occurring in the valence band evolved a number of O_2_^•—^, which could attack the PE chains and trigger the C—C cleavage as well. The authors also demonstrated the universality of this technique that almost complete mineralization of PP and PVC into CO_2_ and H_2_O were achieved within 60 and 90 h, delivering CO_2_ evolution rate of 14.9 and 10.1 mg g_cat_^−1^ h^−1^, respectively (Figure 14b). Apart from the C—C cleavage to mineralize plastics into CO_2_, the C—C coupling occurred in a sequential reactor, in which ^•^COOH intermediates generated via photogenerated electrons triggered CO_2_ reduction resulted in the formation of CH_3_COOH. As the result, the formation rate of CH_3_COOH from PE, PP, and PVC were 47.4, 40.6, and 39.5 µg g_cat_^−1^ h^−1^ (Figure 14b), respectively, which were significantly lower than the CO_2_ evolution rate. DFT calculations were conducted to give insight to the low CH_3_COOH evolution efficiency. It was indicated that long Nb—O bond length resulted in the weak absorption of HOOC—CO· intermediates on the surface of catalyst, which restrained the following reactions for the conversion into CH_3_COOH. Even worse, the endothermic character and large activation energy to overcome the reaction barrier of the formation of ^•^O intermediate (such as, HOOC—CO·), as well as desorption of CH_3_COOH on Nb_2_O_5_ atomic layers, resulted in the unfavorable generation rate of CH_3_COOH.

Uekert et al. reported a photoreforming process to transform waste PET bottles, polylactic acid (PLA) or polyurethane (PUR) into organic small molecules. Taking PET as an example, the reaction was conducted in a 10 m aq NaOH solution at ambient temperature and pressure with the presence of CdS/CdO*_x_* quantum dots (QDs) as catalyst.^[^ ^208^ ^]^ Driven by simulated sunlight, the photoexcited electrons and holes were generated on CdS/CdO*_x_* QDs, which served as active species to reduce H_2_O into H_2_ and to oxidize the waste PET into small organic molecules, that is, formate, acetate, and pyruvate, respectively (Figure 14e). H_2_ generation from water rather than the plastics was confirmed by the mass spectrometry in deuterated solvents. Somehow, pre‐treatment of the plastic substrate by alkaline treatment in dark played a critical role to derive high yield of H_2_ (Figure 14f), which is assumed to be related to the hydrolysis of these polyester waste to form a certain percentage of small molecular weight intermediates which are ready to be degraded by photo‐catalytic process. The authors verified that these oxidation products mainly come from the aliphatic components, which are ethylene glycol units of PET. As another hydrolysis product of PET, TPA was rather stable under the reaction conditions and could be separated and reused for various applications. To avoid using the toxic CdS, Uekert et al. attempted to use the non‐toxic carbon nitride/nickel phosphide (CN*_x_* |Ni_2_P) photocatalyst via a similar protocol as demonstrated in a separation publication.^[^ ^209^ ^]^ Similar to the result using CdS/CdO*_x_* QDs as catalyst, H_2_ and some valuable organic chemicals such as, acetate and formate were detected as the degradation products (Figure 14g). Interestingly, no ^•^OH radical signal (TPA‐OH) was detected in this system after 20 h photoreforming (Figure 14h), which was contrary to the common belief that ^•^OH radicals play the major role during the photocatalytic degradation process. This result was supported by previous reports that suggested the oxidation of photogenerated holes on CN*_x_* were not enough for ^•^OH evolution. This photocatalytic conversion system also exhibited the feasibility for long‐term photoreforming of polyester microfibers and PET bottles, as well as, upscaled photoreforming of polyester microfibers. Nevertheless, the efficiency of H_2_ evolution through the photoreforming of plastic wastes remain low compared to the well‐established technologies of steam reforming of fossil fuels (80–90% conversion) or gasification of plastic (65–95% conversion),^[^ ^210^, ^211^ ^]^ and the selectivity of small organic molecules are also needed to be further controlled. Yet, these examples are valuable in proposing different plastic recycling pathways, where the oxidation pathway through direct holes transfer from the photocatalyst to the plastics put forward a new opportunity for utilizing plastic wastes as carbon sources for valuable organic chemicals recovery. Meanwhile, the photoreforming is meaningful to circumvent the emission of worthless CO_2_ during the traditional photocatalytic mineralization.

### 4.8 Biotechnology

Biotechnology has always been a highly important technique to remedy the environment, both for water bodies and soil. For instance, natural or modified enzymes and microorganisms have been widely used for the application of biomass conversion, nitrogen fixation and water treatment.^[^ ^212^, ^213^, ^214^, ^215^ ^]^ The intake and metabolism of pollutants by microorganism or directly interaction between pollutants and extracellular enzyme of microorganism give rise to the mineralization or transformation of hazardous pollutants into CO_2_, H_2_O, or other non‐toxic small organic molecules.^[^ ^22^ ^]^ With the merits of good efficiency, low operating cost, and no secondary pollution, controlling the pollutants in environment by biotechnology is undoubtedly a development trend in the future. In recent years, biotechnology has also been considered to degrade or recycle the plastic wastes. Geresh et al. successfully isolated a thermophilic bacterium *Brevibacillus borstelensis* from soil and investigated its performance for degrading PE.^[^ ^216^ ^]^ During the biodegradation performed by incubating in soil at 50 °C, PE was utilized by *B. borstelensis* as the sole carbon and energy source. After incubation for 30 d, ≈11% gravimetric weight reduction and ≈30% molecular weight reduction were achieved, respectively. Nevertheless, the degradation rate remains low. To further enhance the degradation speed, external initiators such as, thermal,^[^ ^217^ ^]^ UV radiation,^[^ ^218^ ^]^ and oxidation reagents,^[^ ^219^, ^220^ ^]^ have been employed to facilitate the biodegradation by inducing oxidation and chain scission of polymer chains and form more hydrophilic groups (C = O, —OH, and —COOH), making the substrates more favorable for the adhesion and catalysis of microbes and enzymes.

**Figure 15:**
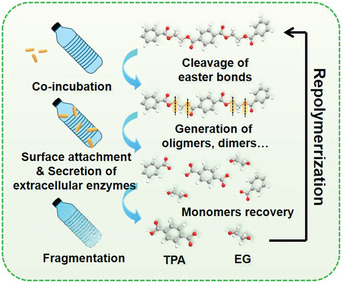
Scheme of biotechnological depolymerization of PET for monomers recovery.

Owing to the presence of hydrolysable ester bonds, biodegradation of PET into monomers is featured as sustainability and economic efficiency (**Figure** **15**). In this context, recycle of PET by biotechnology has emerged. A novel bacterium, *Ideonella sakaiensis* 201‐F6 isolated by Yoshida et al. exhibited the capability to degrade PET.^[^ ^221^ ^]^ The optical photograph and SEM image illustrated the growth of microbial and cavities generated on PET film surface. Under the experiment conditions of performing in a YSV (yeast extract–sodium carbonate–vitamins) medium at pH 7.0 shaken under 300 strokes min^−1^ at 30 °C, PET film was almost completely degraded within 6 weeks. The *ISF6_4831* and *ISF6_0224* proteins were identified as the key proteins to hydrolyze PET into mono(2‐hydroxyethyl) terephthalic acid (MHET) and subsequently into TPA. Additionally, leaf‐branch compost cutinase (LCC) has also been reported as promising enzymes for PET depolymerization. Unfortunately, the high crystallinity of PET negatively affects accessibility of LCC enzyme and consequently leads to sluggish degradation kinetics. Softening PET by heating makes it more easily for enzymes to interact with the polymers. However, a higher incubation temperature of 65 °C leads to the inactivation of LCC enzyme. In this context, Marty et al. modified LCC enzyme by enzyme engineering to enhance its thermal stability through mutagenizing the active sites and replacing the divalent‐metal‐binding site with a disulfide bridge.^[^ ^222^ ^]^ The *WCCG* and *ICCG* variants with excellent specific activity at higher melting temperature were finally selected. As the consequence, remarkably improved PET depolymerization efficiency (≈90%) of this enzyme was achieved after 10 h's incubation under agitation at 72 °C and pH 8.0. Moreover, the recycled high purity TPA monomers (>99.8%) can be utilized to synthesize PET and further process into bottles, which is in accordance with the concept of a circular PET economy.

### 4.9 Overview of Representative Recycling Strategies

**Figure 16:**
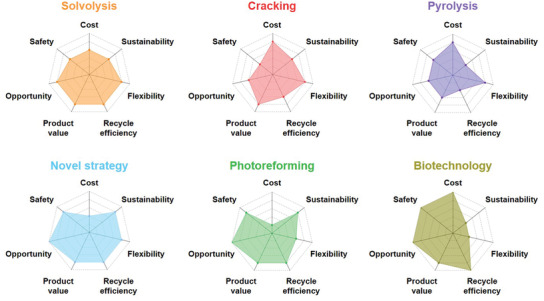
Comparison of the general performance metrics for current plastics recycling strategies.

The development of rational strategies for plastics recycling is meaningful to avoid the mismanaged plastic wastes releasing secondary MPs into nature environment. To give a comprehensive view of the current plastics recycling strategies, the general performance metrics including cost, safety, flexibility, opportunity, operability, product value, and recycle efficiency for representative recycling strategies are summarized and compared in **Figure** **16**. Among these metrics, cost, safety, flexibility, sustainability, recycle efficiency, and opportunity are defined by similar criteria mentioned in Section 3.4. Besides that, product value is to estimate that which recycle categories (closed‐loop recycling, upgrade recycling, or downgrade recycling) the strategies belonging to.

Solvolysis is the most well‐established and reliable method to recycle PET wastes with high selectivity and conversion efficiency, where high purity monomers or other fine chemicals can be regenerated and further utilized as raw materials for polymerization or additives. For PET depolymerization process, glycolysis is thus far the most successful route, which enables the efficient recovery of BHET for PET repolymerization. Ionic liquids and magnetic heterogeneous catalysts based on transition metals are encouraged to pay more attention because of they have shown high selectivity and conversion efficiency, as well as, potential possibility of facile separation. In the future development, advanced catalyst should be further investigate to achieve the mild reaction condition, faster depolymerization kinetic, higher BHET selectivity, easier catalysts separation from final products, and more stable performance for long run. Meanwhile, the development of green cosolvents to overcome the immiscibility of PET with the polyols during the glycolysis is an effective route to enhance the depolymerization kinetics and achieve the fast BHET recovery within minutes.

Cracking is an irreplaceable solution to convert polyolefins into fuels. Nevertheless, the relative high temperature and random scission of C—C bonds give rise to the high energy consumption and low product selectivity, which limit the industrial application. Although C can be operated at lower temperature of ≈200 °C, the high H_2_ pressure of 1–10 MPa is a safety concern. The optimization the interaction between catalyst and molecular chain by interfacial engineering is the potential route to save energy consumption and enhance the product selectivity.

Synthesizing valuable functional carbons derived from plastic wastes via pyrolysis is a rational strategy, which can be used as critical materials in the applications of contaminations absorption, energy storage and conversion. Even though pyrolysis is universally valid for almost all categories of plastics, the higher temperature (>500 °C) and the use of extra reagents (template or activating agent) increase the operation cost. In addition, low carbon yield should be overcome to increase the utilization of precursors.

When combining the emerging techniques (microwave irradiation, plasma treatment, and ROSs treatment) with conventional approaches, it is possible to optimize the conversion pathways toward higher product selectivity and even can save energy input. Future investigations should also focus on studying the interaction between catalyst and plastics or intermediates to further enhance the product selectivity and boost the catalytic conversion efficiency.

Photoreforming opens the avenue of converting plastic wastes into useful fine chemicals under mild condition. The utilization of sunlight as energy source can greatly save energy input. In addition, it is more meaningful to convert the fossil derived plastics into value‐added products rather than simply degrading and mineralizing them into greenhouse gas CO_2_. Though the prospect of photoreforming is promising, the development is in the infancy and the productivity remains low.

Taking the advantages of biotechnology into account, the high conversion efficiency, and environmental friendly final products make it one of the most green and promising strategies to efficiently degrade or recycle plastics without introducing secondary pollution. Nevertheless, there are several challenges should be overcome to achieve the large‐scale application: 1) The complex gene modification to enhance recycling efficiency and stability is an expensive and time‐consuming process; 2) the highly specific of enzyme catalyzed reaction limits the recycling efficiency of direct treating a mixture containing several categories of plastics; 3) the adaptation of microorganism in various environments such as temperature and atmosphere is unknown.

In summary, the emergence of advanced technologies such as microwave‐coupling pyrolysis, photoreforming, and biotechnology possess the potential to overcome the technical bottleneck of high energy consumption, low productivity, and low selectivity. Future research will focus on the rational design of recycling system and catalysts to improve the conversion efficiency and selectivity. There is a long path ahead to develop the fast, efficient, mild, and environmentally friendly recycling technologies for large scale production applications.

## 5 Feasibility Prediction of Backbone Cleavage of Typical Microplastics

As widely known by researchers, the high chemical stability of the saturated backbone of plastics (such as, PE, PP, PVC, PS) endows their excellent resistance to degradation from environmental factors. Generally, a complete degradation of the plastic wastes requires hundreds of years. The molecular weight and structure are imperceptibly changed within this timeframe, which are only observed via the naked eye from their color changes and/or the appearance of some random surface cracks or voids under a microscope. Thus, enabling the cleavage of these inert backbones to accelerate the degradation and mineralization processes is a major challenge of the complete harmless elimination of MPs.

### 5.1 Natural Abiotic Degradation Pathways

Even though thermoplastic polyolefins are known for their high sensitivity toward thermal oxidation which arises from the impurities incorporated in the polymer chain during high temperatures processing. For example, thermal oxidative degradation of PE does not occur at appreciable rates at temperatures below 100 °C. Under the ambient environment, the major degradation process is the photoxidation assisted by the presence of sunlight and oxygen.

**Figure 17:**
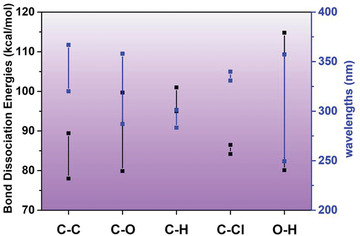
Bond dissociation energies of key chemical bonds and corresponding wavelengths of light.

Overall, the photo‐induced free radical degradation of plastics involves three steps: Initiation, propagation, and termination. Taking account into the key chemical bonds of plastics,^[^ ^223^, ^224^, ^225^, ^226^ ^]^ the bond dissociation energies of C—C, C—O, and C—H, C—Cl, and O—H are 78–89.4, 79.9–99.7, 95–101, 84.2–85.5, and 80.1–114.8 kcal mol^−1^, respectively, which are mostly equivalent to UV radiation from 200 to 400 nm (71.9–96.9 kcal mol^−1^) (**Figure** **17**). In this case, when the input photon energy is higher than the bond dissociation energy in the polymer chain, chemical bond scission is then triggered.

#### 5.1.1 Polyethylene

**Figure 18:**
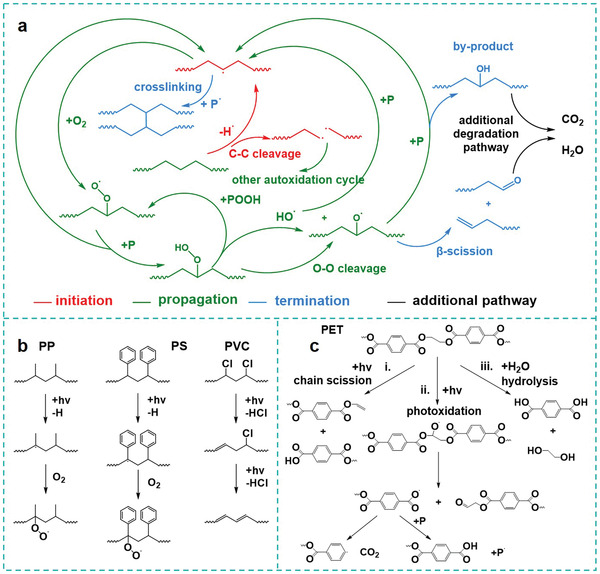
a) Degradation pathways for PE via photo‐induced C—H bond cleavage on the polymer backbone. b) Degradation pathways of PP, PS, and PVC. c) Degradation pathways of PET: i) Direct chain scission; ii) photoxidation; iii) hydrolysis.

PE, possessing a typical inert C—C skeletons and C—H bonds, are mass‐manufactured into short‐life products. The degradation pathway is illustrated in **Figure** **18**a. Initially, when the PE plastics are exposed to UV light (or heat, ROS), the C—H bonds broken in the main polymer chains triggered by hydrogen abstraction lead to the generation of polyethylene alkyl radical (P^•^) and the initiation of degradation. Subsequently, the oxygen is incorporated into the polymer chain by reacting with the alkyl radical to form the peroxy radical (POO^•^). Hydrogen abstraction reaction of POO^•^ with another polymer chain gives rise to the generation of hydroperoxide (POOH) and P^•^, where the former is prone to split into alkyloxy radical (PO^•^) and hydroxyl radical (^•^OH) by the cleavage of the labile O—O bond, the latter can reaction with oxygen and trigger another degradation cycle. It was demonstrated that the activation energy of the *β*−scission of PO^•^ is only 5.7–12 kcal mol^−1^, which is surmountable at ambient temperature.^[^ ^227^ ^]^ Thus, the following decomposition of PO^•^ gives rise to chain cleavage as well as ketones and olefins evolution. Meanwhile, ketone and alcohol can be also generated through the disproportionation of two POO^•^. These oxygen‐containing groups incorporated intermediates are more prone to be degraded, where additional degradation pathways are introduced for further oxidative degradation. These as‐formed oxygen centered radicals all possess strong ability of hydrogen abstraction with another polymer chain to generate P^•^. Although non‐radical hydroxylated polyethylene (by‐products) can be formed by the reaction of alkyloxy radical with polymer, more P^•^ are yielded and serve as the initiators to active more new propagation processes. Besides, C—C bond is possible to be directly broken under UV or heat, where the two P^•^ are generated and play as the initiators of autoxidation cycle. With the continuing of the autoxidation cycle, the long‐chain polyethylene MPs fragments gradually undergo the scission of the backbone and incorporation of oxygen, resulting in the conversion into short‐chain ketone, olefin, and other unsaturated products. During a photo‐induced oxidative degradation, both random chain scission and cross‐linking side reactions are possible, it is predominant to generate lower molecular weight fragments via the former process.^[^ ^191^ ^]^

It is worth noting that the lack of unsaturated chromophoric groups (such as, unsaturated double bonds) in the polymer backbones of polyolefins (especially for PE or PP) prevents them to absorb light energy, which results in the immunization of photo‐induced degradation. However, trace amounts of internal (i.e., structural abnormalities, carbonyl, unsaturated bonds) impurities incorporated into the macromolecular structure during polymerization and storage and external impurities (i.e., organics or inorganics from processing equipment, containers, additives, and surface absorbed substances) permit for photo‐initiated degradation to some extent. For instance, the introduced carbonyl groups in the molecular structure during synthesis or processing can act as the chromophores to accelerate the photolysis through absorbing near UV component of sunlight (280–390 nm). Subsequently, Norrish Type I (radical generation and no chain cleavage) and Norrish Type II (chain cleavage) processes are initiated by the homolytic cleavage of C—C into free radical intermediates and intramolecular *γ*‐H abstraction generates ketones and vinylidenes, respectively. Besides, C = C unsaturated bonds are sensitive sites to react with light generated singlet oxygen (^1^O_2_) by “ene” reactions to form peroxides and start the autoxidation. Furthermore, it is reported that the photoxidation rate of LDPE is higher than HDPE, which can be ascribed to the low density polymer possessing higher frequency of reactive branch points.

#### 5.1.2 Other C—C Backbone Plastics

For other typical plastics with C—C backbone (i.e., PP, PS, and PVC), the photooxidative degradation behaviors mainly depend on their molecular structures (Figure 18b). The photoxidation mechanism of PP plastics is similar to PE, while PP is more prone to be photoxidized owing to the high proportion of lower stable tertiary carbon in the backbone. The subsequent oxygen incorporation and backbone scission result in the formation of oxygen‐containing groups and unsaturated groups, and the reduction of molecular weight. Owing to the presence of phenyl rings in the branches of the backbone, PS is more susceptible to lower the molecular weight and release microplastic fragments in environment. The hydrogen abstraction and following generation of polymer radical can be triggered by the exciting of phenyl ring and the transformation of excitation energy to the nearest C—H bond under UV light. PVC is the most sensitive commonly used polymers under UV radiation. Different from the photoxidation mechanisms of other plastics with C—C backbone, the dechlorination is the first step of PVC degradation under UV radiation, which gives rise to the discoloration of the polymer and the generation of conjugated double bonds along with the release of hydrochloric acid. Due to the autocatalytic character of the dechlorination process, it may continue until there are only traces of chlorine left in the macromolecule. Afterward, the labile unsaturated C = C double bonds are prone to suffer from the photoxidation and cleavage into short chain fragments.

#### 5.1.3 Heteroatomic Backbone Plastics

Taking into account the plastics with heteroatoms in the main chain, PET incorporated by oxygen atoms in the backbone possesses enhanced thermal stability compared to plastics with a solely C—C backbone. Thus, UV light‐initiated photoxidation are the major degradation pathways of PET under ambient conditions (Figure 18c). On one hand, the abstraction of *α*‐H induced direct ester linkages cleavage during the anaerobic photodegradation results in the formation of a vinyl‐terminated chain and a carboxylic acid‐terminated chain. On the other hand, aerobic photoxidation initiated by the *α*‐H abstraction of ester also gives rise to chain scission, where the mechanism is similar to the plastics with C—C backbone. Briefly, a peroxy radical is generated by the incorporation of oxygen atoms by the reaction between the formed carbon centered radical and oxygen, which can abstract the *α*‐H from another molecular chain to promote the propagation process. Besides, the cleavage of O—O bond of hydroperoxide lead to the generation of hydroxyl radicals, which can attack the aromatic rings in the polymer backbone to form hydroxyterephthalate groups. As a consequence, the rotation of autoxidation cycle gives to the lower of molecular weight and the release of small molecule carboxylic acids, aldehydes, and other oxygenates. Moreover, moisture‐induced hydrolysis is common under ambient conditions as well, which breaks the ester linkage to generate shorter carboxylic acid‐terminated and alcohol‐terminated chains. Ultimately, the complete hydrolysis leads to the formation of TPA and ethylene glycol, where the monomers can be reused for polymerization and returned the polymer industry. Unfortunately, the pH dominated hydrolytic cleavage of PET is very slow in the near‐neutral water body. While in the landfill with insufficient moisture, a more pronounced local drop in pH results from the formation of carboxylic acid‐terminated small molecules can facilitate the ester hydrolysis.

### 5.2 Biodegradation of the Microplastics

In addition to the abiotic degradation, the biodegradation is a significant pathway to eliminate MPs pollution by microorganisms. The nature of biodegradation is chemical process, while the microorganisms are the source of the attacking chemicals. During the biodegradation process, the assimilation by microorganisms or direct degradation by extracellular enzymes can break the polymer backbone and subsequent reduction in the average molecular weight, which leads to the change in surface properties or loss of mechanical strength. However, biodegradation resistance of commercial plastics is high. The long molecular chain and insufficient functional groups make polyolefins are more resistant toward biodegradation. It is worth noting that biodegradability of polymer is connected with the chemical structure. For instance, the tertiary carbon of PP and chlorines of PVC increase the resistance to microbial degradation. In addition, the high crystallinity of PET endow it compact structure, which increases the penetration resistance of moisture and enzymes. Although a lot of research has been carried out on biodegradation, the degradation of plastics in the natural environment still relies on abiotic processes to a certain extent. Thus, the abiotic degradation involving the polymer backbone scission and formation of functional groups is necessary preceding the biodegradation. The increasing carbonyl index leads to increasing of the hydrophilicity of the small polymer fragments formed by abiotic degradation. Therefore, the microbial adhesion toward the debris with hydrophilicity along with high specific surface area can be enhanced, which is more available for biodegradation.

### 5.3 Boosting the Degradation of the Microplastics

The photo‐induced H atom abstraction reaction of saturated polymers has a significant activation energy (typically of the order of 9.6–12 kcal mol^−1^), while oxygen incorporation reaction is driven by the exothermicity with zero activation energy.^[^ ^228^, ^229^, ^230^ ^]^ Therefore, the H absorption process is the rate‐determining step hindering the subsequent propagation process. In order to accelerate the degradation of MPs, the boosted H atom abstraction is a pressing need. ^•^OH is a highly reactive oxygen‐centered radical whom bimolecular rate constants for reaction with organic compounds (hydrogen atom abstraction) approach the diffusion‐controlled limit in aqueous solution. It has been verified that the ^•^OH is able to break the C—H of polymers (i.e., PET, PE, PP, PVC, PS) and thus induces the incorporation of oxygen containing groups and the cleavage of polymer's C—C backbone.^[^ ^108^, ^207^ ^]^ Finding the relationship between the reactivity toward the hydrogen atom abstraction and a molecular property is always useful. Such a relationship can be used to estimate the environmental lifetime of MPs. It can be expected that the reactivity toward the ^•^OH radical will vary systematically for a series of homologous compounds. For polyolefins with aliphatic hydrogens, the per‐hydrogen reactivity is approximately tertiary > secondary > primary, based upon a multiple regression analysis of the results (assuming the global rate constant to be the sum of contributions from each type of hydrogen).^[^ ^231^ ^]^ To facilitate the degradation and mineralization of MPs, more efforts will be attempted to investigate the ^•^OH or other oxidative species (i.e., SO_4_^•−^ and h^+^_VB_) induced degradation behavior of MPs.

### 5.4 Design of Degradable Plastics

Developing degradable plastics that can be fully degraded into environmentally friendly substances in natural environments as alternatives of traditional non‐degradable plastics is an effective way to address the plastic wastes accumulation and persistent MPs issues. In recent years, degradable poly(lactic acid) (PLA), poly(caprolactone) (PCL), and poly(vinyl alcohol) (PVA) plastics have been developed and widely used in biomedical equipment, food packaging, and disposable tableware.^[^ ^232^, ^233^, ^234^, ^235^, ^236^, ^237^ ^]^

Many efforts have been done on the design of rational polymer structures to accelerate the degradation process. DelRe et al. reported a rapid degradation of PLA and PCL by using random heteropolymer (RHP) to nanoscopically incorporate the enzymes into the polymer structure.^[^ ^238^ ^]^ The physical and chemical properties of the modified plastics are not obviously changed. When exposed to heat and water, the enzyme breaks away from the RHP protectant and begins to break down plastics. Both of the as‐designed enzyme‐RHP‐PCL complex and enzyme‐RHP‐PLA delivered accelerated depolymerization in industrial soil composts, which were clearly disintegrated in 2 days at 40 °C and 6 days at 50 °C, respectively. Li et al. designed a PVA‐based supramolecular plastic by the complexation of vanillin‐grafted PVA (VPVA), hydrophobic humic acid (HA), and Fe^3+^ ions (VPVA–HA–Fe complexes), which can be completely degraded within ≈108 d placed under soil without requiring any other manual interference.^[^ ^239^ ^]^ Since the presence of high‐density hydrogen bonds and coordination interactions, the VPVA–HA–Fe complexes delivered excellent breaking strength of ≈85.0 Mpa, which is obviously higher than that of traditional PE plastics (15–30 MPa). At the primary stage of the degradation under soil, the VPVA–HA–Fe plastic gradually absorbed water from the soil and become swelling, which can be ascribed to the partially breakdown of inner hydrogen bonds and coordination interactions within the plastic. Meanwhile, the presence of Fe^3+^, and other inorganic ions in soil (Ca^2+^ and Al^3+^) can serve as Lewis acids catalysts, leading to the sissicion of bond between Vanillin and VPVA chains through hydrolysis. Subsequently, the microorganisms adhere onto the plastic surfaces and secrete enzymes to decompose the PVA chains into non‐toxic small‐molecular species. At present, the mechanism of degradable plastic is mainly based on esterase produced by microorganisms to trigger the cleavage of ester bond on the polymer chain. However, the degradation efficiency highly depends on plastics’ surrounding environment (i.e., microorganisms, enzymes, pH value, temperature, and humidity), which greatly limits their degradation in the natural environment. Tian et al. designed a novel degradable conjugated polymer poly(deca‐4,6‐diynedioic acid) (PDDA), which can make full use of the physical and chemical factors in the natural environment to generate non‐toxic substances without relying on biodegradation.^[^ ^240^ ^]^ PDDA delivered self‐degradation efficiency of fully decomposition within a week through photooxidation with the presence of sunlight and air, while the chemical composition and physicity properties of PDDA are stable in the dark. The authors also covered the degradation of PDDA could be ascribed to the photo‐induced cleavage of C = C and C ≡ C bonds in polymer backbone.

There is no doubt that the advent of degradable plastics opens up new opportunities to mitigate the long‐term MPs pollution by using novel plastics with rapid degradation kinetics. However, the complete degradation of degradable plastics in extreme condition also take several decades to hundreds of years, which is comparable to non‐degradable counterparts.^[^ ^24^ ^]^ Worse more, the instability of degradable plastics under heat, UV light, and microorganism may result in rapid release of MPs and cause severe short‐term risk. Therefore, the production and disposal of biodegradable plastics need to be strictly regulated. Meanwhile, the mechanism of degradable plastics should be further investigated to enhance the controllability and degradation rate in complex environment.

## 6 Challenges and Perspectives

MPs in the environment are a critical and urgent issue, attracting attention from the public, government, researchers, and funding bodies due to their distribution and potential threat to ecosystems. These innocuous seeming, yet extremely hazardous MPs are prone to be uptake by living organisms and result in irreparable damage to ecological systems. Even more terribly, the hydrophobicity and large specific surface area of MPs make them substrates for heavy metal ions, POPs, and pathogenic bacteria, which impose serious negative impacts on biota and human. Nevertheless, most existing strategies applied in practical application are insufficient to solve the MPs issue, resulting in the rapid accumulation of MPs in the environment. Humanity must overcome these challenges, to develop and implement technology enabling the construction of a MPs‐free environment. In this review, we highlight the current efforts surrounding advanced approaches for catalytic conversion aiming to the MPs elimination and plastics recycling. We hope this review helps generate new ideas for catalytic methods that can overcome barriers discussed throughout.

According to the significant achievements for building a MPs‐free environment in the recent years, we summarize and predict the main prospects in this field are as follows:

1) The development of advanced methods for identification and quantification of MPs is not to distinguish and detect the environmental MPs. More importantly, rational MPs characterization methods are beneficial to give insight into the MPs’ degradation behavior in the natural environment and in the process of catalytic degradation. For example, in situ infrared and in situ Raman measurements can transiently monitor the degradation of MPs from the variation of chemical bonds. Meanwhile, the development of advanced identification and quantification methods is conducive to the investigation of degradation kinetics and product selectivity.
2) Although the existing traditional microplastic removal strategies (absorption, ultrafiltration, and membrane technology) have exhibited certain efficiency, it is of greater significance to investigate chemical methods of MPs to degrade or mineralize MPs into harmless matter such as CO_2_ and H_2_O. Powerful organic pollutants degradation strategies such as photocatalysis and AOPs will play important roles in the future. According to the chemical stability endowed by the chemical structure of most MPs, as well as, the high crystalline of plastics, the synergy of appropriate external energy sources, and reasonable design of the reactors can overcome the intrinsic degradation resistances and accelerate the degradation of MPs. In addition to CO_2_ and H_2_O, it is also possible to develop sustainable strategies to transfer MPs into valuable fine chemicals. Meanwhile, developing of advanced technologies is urgently needed to remove MPs in air and soil, since more and more studies are published to cover the potential risk about these MPs.
3) Inspired by coupling technologies in the application of detection and catalysis, the concept of combination of different technologies have the potential to promote the developing of MPs removal approaches. The synergistic effect of physical–chemical, physical–biological, or chemical–biological coupling approaches may result in the improved degradation efficiency of MPs. For instance, the design of functional membrane with photocatalysis feature and adjustable porosity will deliver excellent MPs removal efficiency by trapping and in situ photocatalytic degradation. Meanwhile, the physical or chemical approaches (i.e., UV light, and AOPs) can be used as the pre‐treatment to reduce the molecular weight and enhance the hydrophilicity of MPs by backbone cleavage and oxygen incorporation, which is essential to promote the following biodegradation process. To achieve the better synergistic effect, more considerations should be given to avoid the mutual interference between the two technologies.
4) In addition to the thorough harmless treatment methods of environmental MPs, significance should be attached to intentional degradation and recycling of bulk plastic wastes. Reasonable strategies for plastic waste recycling can efficiently reduce the important source of secondary MPs. Compared with the weathered microplastic fragments in natural environment, bulk plastic wastes that exist for a short time in nature are easy to collect and are relatively pure, which are more facile and valuable to recycle. Moreover, developing efficient method for sorting, separation and purification of plastics from the mixed waste steams is of great significance in practical application, since the presence of impurities (including other plastics and additives) can reduce conversion efficiency generate undesired products and damage the equipment.
5) Catalysts act in an important role in the conversion of plastics into valuable products. In the future, the rational design of dual‐functional site catalysts based on the interfacial engineering to selectively adsorb certain intermediates and following in situ catalytic transformation is essential to adjust the selectivity of product.
6) Beyond the stereotype of traditional recycling methods, we should reconsider and innovate the recycling of plastic wastes through the frontier technologies such microwave, plasma, and AOPs, and so on. Through process engineering enabled by frontier technologies, the reaction pathways and products are controllable to yield more valuable products. As traditional recovery strategies are dominated by thermal treatments, the heating and cooling of the reactors cause a large amount of heat waste, which is not in line with the concept of sustainable development. Therefore, the utilization of frontier technologies to act as external energy sources or reduce the activation energy of the reaction is a meaningful strategy to alternate the traditional high temperature heat. Ultimately, the most ideal recycling method will tend to mild conditions, facility and economy.
7) It's worth noting that there is a massive amount of plastic wastes (≈4900 Mt) degrading and releasing MPs in landfill and natural environment already. Therefore, collecting and recycling the plastic wastes from landfill and developing technologies as well as catalysts that can function under various environments (i.e., underground without light, deep‐sea under low temperature, or other condition in the presence of anions, heavy metal ions, POPs, and microorganisms) to stop the generation of MPs is critical. Otherwise we're stopping the problem from exponentially getting worse—but not actually making it better.
8) The design of the degradable polymer is also very critical. On the premise of not affecting performance, some inorganic or organic motifs can be introduced in the polymer framework as the switch of degradation to induce and accelerate the degradation of waste plastics in certain condition. For instance, the introduction of inorganic matters with high expansion coefficient is capable to destruct the compact high crystalline structure, so that the degradation of waste plastics can be accelerated by the enhanced accessibility with the presence of UV or oxidation reagents. More importantly, the production and disposal of biodegradable plastics need to be strictly regulated. Otherwise, it will worsen short‐term MPs pollution, since the degradable polymers possess more rapid MPs generation.

## Conflict of Interest

The authors declare no conflict of interest.
